# Preparation and Preclinical Characterization of a Simple Ester for Dual Exogenous Supply of Lactate and Beta-hydroxybutyrate

**DOI:** 10.1021/acs.jafc.4c04849

**Published:** 2024-08-30

**Authors:** Rasmus N. Ottosen, Jacob M. Seefeldt, Jakob Hansen, Roni Nielsen, Niels Møller, Mogens Johannsen, Thomas B. Poulsen

**Affiliations:** † Department of Chemistry, Aarhus University, Langelandsgade 140, Aarhus C DK-8000, Denmark; ‡ Department of Cardiology, Aarhus University Hospital, Palle Juul-Jensens Boulevard 99, Aarhus N DK-8200, Denmark; § Department of Forensic Medicine, Aarhus University, Palle Juul-Jensens Boulevard. 99, Aarhus N DK-8200, Denmark; ∥ Steno Diabetes Center Aarhus, Aarhus University Hospital, Palle Juul-Jensens Boulevard 11, Aarhus N DK-8200, Denmark; ⊥ Department of Clinical Medicine, Aarhus University, Palle Juul-Jensens Boulevard 82, Aarhus N DK-8200, Denmark

**Keywords:** lactate, ketones, exercise mimetics, exerkine, *N*-L-lactoyl-phenylalanine (Lac-Phe), appetite regulation

## Abstract

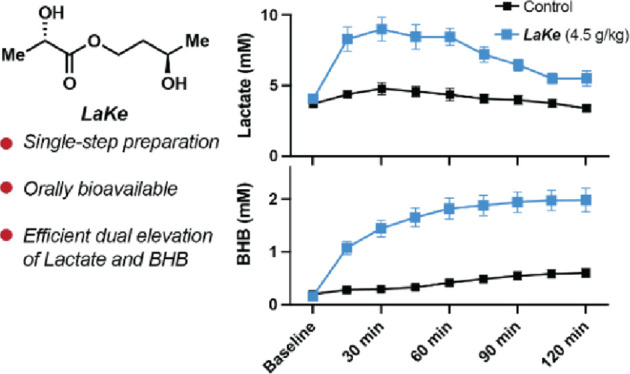

Elevation of the plasma levels of (*S*)-lactate (Lac) and/or (*R*)-beta-hydroxybutyrate (BHB) occurs naturally in response to strenuous exercise and prolonged fasting, respectively, resulting in millimolar concentrations of these two metabolites. It is increasingly appreciated that Lac and BHB have wide-ranging beneficial physiological effects, suggesting that novel nutritional solutions, compatible with high-level and/or sustained consumption, which allow direct control of plasma levels of Lac and BHB, are of strong interest. In this study, we present a molecular hybrid between (*S*)-lactate and the BHB-precursor (*R*)-1,3-butanediol in the form of a simple ester referred to as ***LaKe***. We show that ***LaKe*** can be readily prepared on the kilogram scale and undergoes rapid hydrolytic conversion under a variety of physiological conditions to release its two constituents. Oral ingestion of ***LaKe***, in rats, resulted in dose-dependent elevation of plasma levels of Lac and BHB triggering expected physiological responses such as reduced lipolysis and elevation of the appetite-suppressing compound *N*-L-lactoyl-phenylalanine (Lac-Phe).

## Introduction

Exercise is, together with caloric restriction, widely recognized as the most powerful intervention increasing healthy lifespan by preserving muscle mass and cognitive functions. Extensive research efforts have been dedicated to unraveling the physiological response triggered by exercise, with a particular focus on identifying the metabolic drivers behind its adaptive and protective effects.^1−5^ Among the noteworthy findings, a remarkable surge in circulating lactate levels has been observed, transitioning from micromolar to millimolar within minutes of engaging in physical activity.^6,7^

Traditionally regarded as a metabolic waste product, lactate, a small alpha-hydroxylated organic acid, has recently emerged as a significant mitochondrial fuel and circulating messenger.^8,9^ This shift in understanding has been facilitated by the discovery of its interaction with G-protein-coupled receptor HCAR1, along with various alternative signaling mechanisms. Consequently, it is now hypothesized that lactate may serve as a key exerkine,^10−12^ mediating numerous physiological effects of exercise on human physiology.^4,12−16^

Supporting this hypothesis, a multitude of preclinical and clinical investigations have demonstrated that exogenous lactate administration elicits biological responses comparable to those observed during exercise. Such responses include the elevation of crucial hormones, such as BDNF, VEGF, LEAP2, GLP-1, Lac-Phe, as well as other significant physiological reactions.^16−23^

In parallel, ketone bodies, beta-hydroxybutyrate (BHB) and acetoacetate, exhibit a noteworthy increase from micromolar to millimolar levels during exercise, albeit at a more modest pace as glycogen stores are depleted.^6,24^ During caloric restriction or fasting, ketones rise to a similar extent.^25^ Similar to lactate, ketone bodies were also traditionally considered undesired metabolic byproducts, although by now, it is well-established that they not only serve as excellent mitochondrial fuels but also function as vital cellular messengers. Their signaling occurs through the distinctive G-protein-coupled receptor HCAR2, along with alternative mechanisms, thereby impacting positively on a number of aging-related diseases.^25−29^ Clinical studies employing exogenous ketone esters or salts have substantiated their efficacy in inducing these effects.^30−32^

Intriguingly, a number of preclinical and clinical trials have reported strikingly beneficial effects of both lactate and ketones on, for example, cardiac performance^20,32,33^ and appetite regulation,^21,22,34^ suggesting a possibility for additive or synergistic effects of the two compounds in tandem. Such aspects are perhaps particularly compelling in brain aging and disease.^35,36^ This is supported by a high number of preclinical studies demonstrating how exogenous lactate appears to favorably impact brain homeostasis and depression,^37^ an effect potentially mediated via stimulation of neurogenesis/angiogenesis.^19,38−43^ Ketone bodies, in comparison, seem to exert neuroprotective effects and thereby stimulating cognitive functions.^44−46^

Consequently, there is growing interest in the development of new nutritional solutions that enable safe, dual supplementation of lactate and beta-hydroxybutyrate (BHB)/acetoacetate (AcAcO). Ideally, this would involve structurally simple and orally bioavailable compounds that can efficiently release both metabolites, or their direct precursors, while being devoid of any additional byproducts, thereby allowing long-term consumption.

## Materials and Methods

### Synthesis Chemistry: General Methods

Reactions were conducted in flame-dried glassware under an atmosphere of dry argon, unless otherwise stated. THF was dried over aluminum oxide via an MBraun SPS-800 solvent purification system. MeOH and DMF was purchased as anhydrous and further dried and stored under argon over activated molecular sieves (4 Å). The dryness of the solvents was controlled via Karl Fischer titration. Other reagents were used as received from commercial suppliers, unless otherwise stated. DIPEA was dried by stirring for at least 30 min over CaH_2_ followed by distillation onto preactivated molecular sieves (4 Å). Concentration in vacuo was performed using a rotary evaporator with the water bath temperature at 35 °C, followed by further concentration using a high vacuum pump. TLC analysis was carried out on silica-coated aluminum foil plates (Merck Kieselgel 60 F_254_). The TLC plates were visualized by UV irradiation and/or by staining with KMnO_4_ stain (KMnO_4_ (5.0 g), 5% NaOH (aq., 8.3 mL), and K_2_CO_3_ (33.3 g) in H_2_O (500 mL)). Molecular sieves were activated by drying in the oven at 120 °C for at least 24 h, before they were heated in a microwave at maximum power for 2 min, followed by evaporation of the formed vapor on a high vacuum line. This was repeated three to four times and finished by gently flame-drying the flask containing the molecular sieves. Automated flash column chromatography (AFCC) was carried out with an Interchim PuriFlash 420 or Interchim PuriFlash 5.050 using 30 μm prepacked columns. Mass spectra (HRMS) were recorded on a Bruker Daltonics MicrOTOF time-of-flight spectrometer with positive electrospray ionization. Nuclear magnetic resonance (NMR) spectra were recorded on a Varian Mercury 400 MHz spectrometer running at 400 and 101 MHz for ^1^H and ^13^C, respectively. Chemical shifts (δ) are reported in ppm relative to the residual solvent signals (CDCl_3_: 7.26 ppm ^1^H NMR, 77.16 ppm ^13^C NMR, etc.). Multiplicities are indicated using the following abbreviations: s = singlet, d = doublet, t = triplet, q = quartet, h = heptet, and m = multiplet.

For remaining methods (all synthetic procedures and characterization data), see the Supporting Information.

### Stability of ***LaKe*** upon Storage

This experiment was carried out at NCK A/S.

Following preparation, ***LaKe*** was analyzed by GC-FID. Subsequently, ***LaKe*** was stored neat at either −15, 0, and 20 °C and the samples were reanalyzed after 24 months. Table S1 (Supporting Information) contains the corresponding stability data and the GC-FID conditions applied.

### In Vitro Stability of ***LaKe***

These experiments were carried out at Wuxi AppTec.

The in vitro stability of ***LaKe*** was evaluated following incubation in simulated gastric fluid (SGF), simulated intestinal fluid (SIF), fresh and frozen plasma (rats and humans, respectively), and the presence of hepatocytes (rats and humans) and liver microsomes (rats and humans). At the chosen time points, the mixtures were analyzed by LC-MS/MS to quantify the remaining ***LaKe*** and all assays included at least one control compound with known pharmacokinetic properties.

For detailed procedures for these experiments, see the Supporting Information.

### Short-Term Pharmacokinetics of ***LaKe*** in Rats

This method was used to generate the data presented in Figures 6 and 8.

Male Sprague–Dawley rats (300–350g, Taconic, Ry, Denmark) were kept for acclimatization at a constant temperature of 23 °C with a 12 h light-dark cycle and with unlimited access to food and water. All animal handling was in accordance with national guidelines in Denmark and the guidelines from the Directive 2010/63/EU of the European Parliament on the protection of animals used for scientific purposes, and all experiments conformed to Danish Law (Act. No. 1306 of 23/11/2007). The experimental protocol was approved by the Danish Animal Expectorate (2018-15-0201-01475).

The rats were randomly selected to receive either ***LaKe*** (*n* = 8) or placebo (*n* = 8).

Rats were anesthetized in an induction chamber with 8% Sevoflurane (Sevorane, AbbVIE A/S, Copenhagen, Denmark) mixed with oxygen saturated atmospheric air (flow: 2.0 L/min). Upon achieved anesthesia, the rats were intubated and connected to a mechanical ventilator (Ugo Basile 7025 rodent ventilator, Comerio, Varese, Italy) with an adjusted flow of 1.0L/min with 3.5% Sevoflurane. Body temperature was kept at a constant of 37 ± 1 °C with a temperature probe (UNO, Zevenaar, Holland). A PTFE-coated flexible orogastric tube (Fuchigami, Japan) was placed, and the rat was left for stabilization for 15 min.

After stabilization, a baseline blood sample was collected from the rat tail vein before administration of ***LaKe*** or placebo. ***LaKe*** was diluted in physiological saline solution (0.9% NaCl) and administered as a single bolus of 2 mL, per oral dose (4500 mg/kg). Placebo animals received a single bolus of 2 mL of physiological saline solution (0.9% NaCl). Following the baseline blood sample, a bolus of ***LaKe***-solution or placebo was administered via the orogastric tube to the animals. Every 15 min for a period of 2 h, blood samples (200 μL each) were collected in microvettes (sarstedt, 20.1280.100) and were left to coagulate for 30 min followed by centrifugation at 4 and 1500 °C for 20 min. Serum was collected and stored at −80 °C for further analysis by LC-MS/MS (BHB, lactate, and *N*-L-lactoyl phenylalanine (Lac-Phe)) and for nonesterified free fatty acids (FFA).

For detailed procedures for LC-MS/MS and FFA analyses, see the Supporting Information.

### Statistical Analyses

Data are expressed as mean ± SD unless otherwise specified. *P*-values <0.05 were considered statistically significant. Based on our own experience and reports by other research groups, a sample size of minimum *N* = 6 was considered adequate to identify a treatment effect, unless specifically stated. Experiments were performed with parallel administration of intervention and control solutions to eliminate potential time effects. Rats were randomly selected to receive either ***LaKe*** or the control solutions. The effect of two categorial variables, time and randomization group, on a dependent variable (plasma concentration of selected metabolites) was evaluated using a mixed-effect model for repeated measures. Bonferroni’s correction was applied for multiple comparisons. All analyses were performed using GraphPad Prism 8.2.0 (Graph Pad Software, CA, USA).

### Extended Pharmacokinetics of ***LaKe*** in Rats

This experiment was carried out at Wuxi AppTec. This method was used to generate the data presented in Figure 7.

Animals (SD rats, both male and female, 7–9 weeks) were individually housed from 24 h before dosing until the end of the study. Food was withdrawn the night before initiation of study; following food withdrawal, animals were maintained on glucose water containing 5% w/v glucose and 1% saline to suppress endogenous ketone production throughout the study. Food was returned 8 h post-test article dosing. Animals were monitored for distress or notable changes in behavior during the study. The animals were euthanized by CO_2_ inhalation 2 h after the last sample collection time point.

Blood collection was performed by jugular vein cannulation of each animal and divided into two parts. About 10–15 μL of blood was used for the analysis of glucose by glucometer measurement. About 0.20 mL of blood was collected into prechilled commercial EDTA-K2 tubes containing 5 μL of the esterase inhibitor (100 mM PMSF in ethanol, 40 mg/mL NaF and 120 mg/mL KO in water = 5:95(v/v). All blood samples were placed on wet ice until centrifugation.

At least 100 μL plasma was collected respectively and transferred into polypropylene tubes with 400 mM TTFA (2-thenoyltrifluoroacetone) in ethanol (plasma:stabilizer = 100:5 (v:v)). The final concentration of TTFA in the plasma sample from each time point was approximately 20 mM. The samples were snap frozen over dry ice and kept at −60 °C or lower until LC-MS/MS analysis for 3-hydroxybutyl 2-hydroxypropanoate (***LaKe***), lactate, and β-hydroxybutyrate (BHB).

For detailed procedures for LC-MS/MS-analyses, see the Supporting Information.

## Results and Discussion

### Design of ***LaKe***

**Figure 1:**
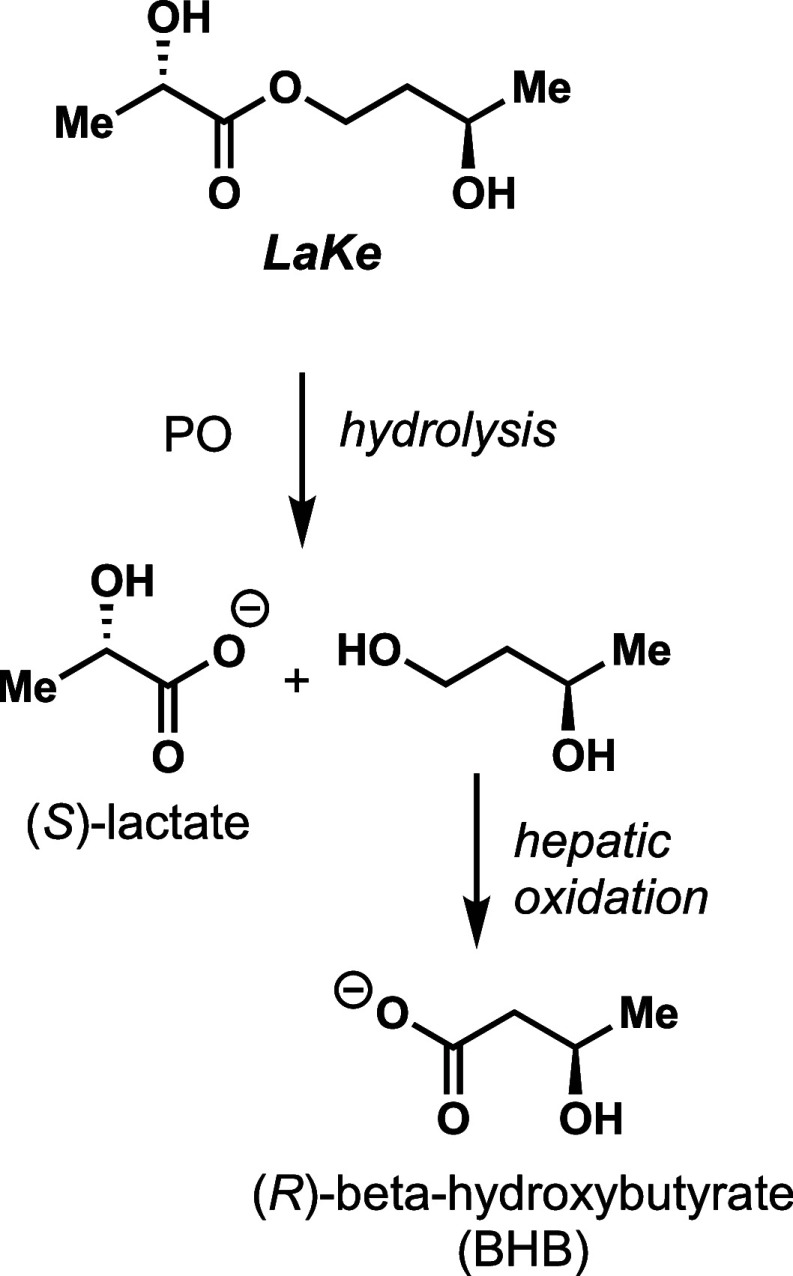
Design of ***LaKe***. A simple, neutral ester that allows hydrolytic release of (*S*)-lactate and, following subsequent oxidation, (*R*)-beta-hydroxybutyrate (BHB).

We considered several different designs to achieve dual oral delivery of lactate and BHB and identified the primary carboxylic ester of (*S*)-lactate and (*R*)-1,3-butanediol as possibly the simplest solution (Figure 1). We hypothesized that this molecule, which we refer to as ***LaKe***, would be hydrolytically cleaved, either upon exposure to pH extremes or, more likely, via the enzymatic activity of hydrolases (lipases) that are abundant in the gastrointestinal tract and in plasma. Hydrolysis of the ester bond would liberate (*S*)-lactate and (*R*)-1,3-butanediol directly, the latter being a well-known precursor for BHB/AcAcO following oxidation in the liver (Figure 1).^47^ The release of these two molecules were particularly appealing from a safety perspective: (*S*)-lactate is a natural metabolite and (*R*)-1,3-butanediol is generally recognized as safe (GRAS)^48^ for large-scale human ingestion and the same building block also features in the design of pure ketone esters, such as (*R*)-3-hydroxybutyl (*R*)-3-hydroxybutyrate^47^ and bis-hexanoyl (*R*)-1,3-butanediol.^49^ By formation of the (neutral) ester, the use of salt-forms is bypassed. This is a critical parameter as continuous ingestion of large quantities of, for example, the sodium or potassium salts of (*S*)-lactate and (*R*)-BHB/AcAcO risk exceeding the safe dose recommended by authorities and may cause electrolyte imbalances and gastrointestinal side-effects.^50^ Such concerns would be even more pronounced in weakened individuals and people with reduced organ function, for example, of the kidneys. We also considered hybrid lactate-ketone designs featuring different smaller oligomers of lactate (as polylactate is a biocompatible polymer); however, the resulting congested esters might be subject to much slower hydrolysis and thereby induce elevated plasma concentrations of synthetic compounds with unknown properties or long-term effects. In conclusion, ***LaKe*** seemed like an optimal and simple design, subjected to the conditions that the compound could be readily prepared on large scale and that it would be rapidly converted to its constituents.

### Chemical and Chemoenzymatic Synthesis of ***LaKe***

**Figure 2:**
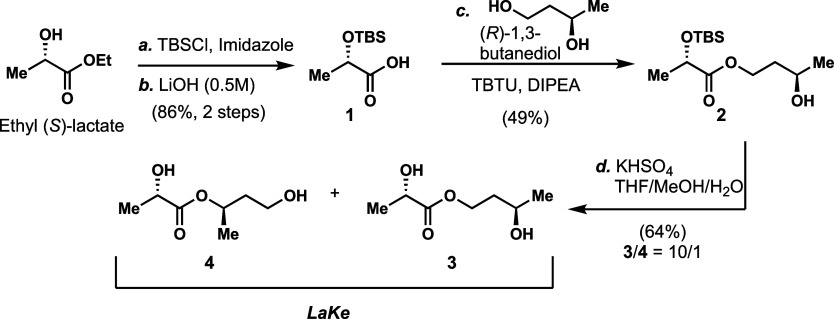
Gram-scale, chemical synthesis of ***LaKe*** from ethyl (*S*)-lactate and (*R*)-1,3-butanediol. Reagents and conditions: (a) TBSCl (1.5 equiv), imidazole (3.5 equiv), DMF, rt, 3 h; (b) LiOH (0.5 M aq., 2.0 equiv), THF, 0 °C to rt, 5 h; (c) TBTU (1.5 equiv), DIPEA (2.1 equiv), DMF, rt, 1 h then (*R*)-1,3-butanediol (1.0 equiv), rt, 16 h; (d) KHSO_4_ (0.25 equiv), THF/H_2_O/MeOH (10/10/4), rt, 16 h. Abbreviations: TBTU = 2-(1*H*-benzotriazole-1-yl)-1,1,3,3-tetramethylaminium tetrafluoroborate; TBS = *tert*-butyldimethylsilyl; DIPEA = *N,N*-diisopropylethylamine; THF = tetrahydrofuran.

We first developed a short chemical synthesis of the structure (Figure 2). Toward this end, we started with commercially available ethyl (*S*)-lactate. Following standard protection of the secondary hydroxyl group as a *tert*-butyldimethylsilyl (TBS) ether, the ester was hydrolyzed in the presence of aqueous sodium hydroxide solution. The resulting acid (**1**) was subjected to ester coupling with (*R*)-1,3-butanediol to selectively afford primary ester **2**. Finally, we removed the TBS group and, following some experimentation, found that this was best accomplished in the presence of the weak acid KHSO_4_ in a mixed solvent system. We used normal column chromatography on silica gel to purify the compound, which by NMR analysis was found to comprise a 10/1 mixture of the primary (**3**) and secondary (**4**) ester. Given the obvious ability of **3** and **4** to interconvert via an intramolecular transesterification (vide infra), we speculated that some degree of equilibration occurred during the deprotection or subsequent purification. Subsequently, we refer to any mixture of **3** and **4** as ***LaKe***. Our synthesis could readily deliver ***LaKe*** on a multigram scale. Furthermore, we also demonstrated that the same overall sequence could be used to afford a stereochemical isomer made from (*R*)-lactate and (*R*)-1,3-butanediol (see the Supporting Information), thereby offering an alternative possibility for delivery of (*R*)-lactate. Indeed, this overall sequence is compatible with any stereoisomeric form and, thereby, also with racemic starting materials.

**Figure 3:**
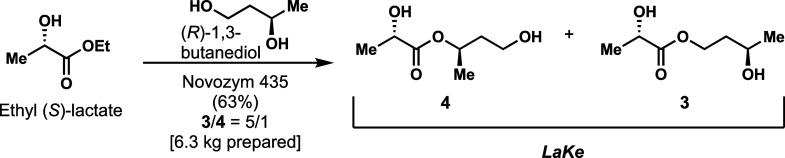
Kilogram-scale, chemoenzymatic synthesis of ***LaKe***.

Next, we sought to simplify the approach substantially. Lipase enzymes are known to catalyze transesterification reactions with high efficiency.^51^ We therefore evaluated whether direct, single-step preparation of ***LaKe*** could be performed from ethyl (*S*)-lactate and (*R*)-1,3-butanediol. Indeed, following optimization of several parameters, we were able to conduct a very efficient synthesis in the presence of immobilized Novozym 435 with 5 equiv of ethyl (*S*)-lactate. This process was readily scalable, and through final purification by distillation, we prepared 6.3 kg corresponding to a 63% yield (Figure 3).

**Figure 4:**
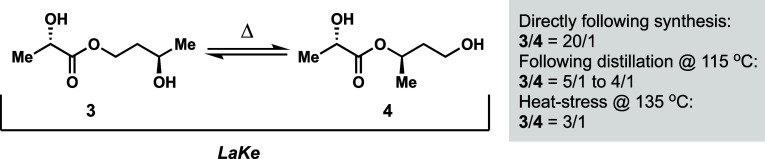
Chemical equilibrium between primary ester (**3**) and secondary ester (**4**).

As in the case of the chemical synthesis, we again observed a mixture (5/1) of **3** and **4**. Systematic study showed that **3** was the exclusive product directly following the synthesis; however, by heating the sample in relation to the distillation (115 °C) or by further heating (135 °C), the ratio of **3** to **4** approaches 3/1 (Figure 4). All in all, we developed different synthetic methods to access ***LaKe***. The chemoenzymatic approach is operationally extremely simple, allowing us to readily proceed to kilogram scale. The compound showed stability to storage in the neat state for at least 2 years at ambient temperature (Table S1, Supporting Information).

### In Vitro Pharmacokinetics of ***LaKe***

**Figure 5:**
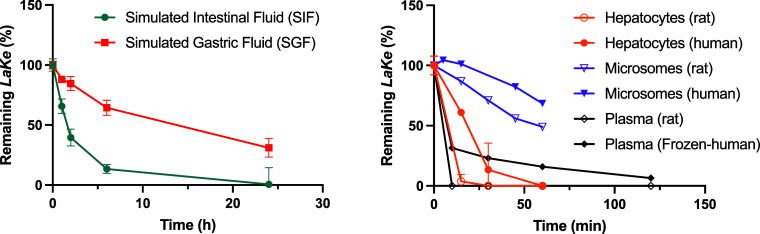
In vitro pharmacokinetics of ***LaKe***. SIF = simulated intestinal fluid. SGF = simulated gastric fluid. SIF/SGF data are mean ± SD (*N* = 2). Hepatocyte data are mean ± SD (*N* = 2). Microsome and plasma data are mean (*N* = 2).

We evaluated the stability of ***LaKe*** under a variety of different conditions (Figure 5). While the compound does undergo conversion under conditions simulating the environment of the stomach (SGF, pH = 1), it was not very rapid (*T*_1/2_ > 12 h). Much faster conversion (*T*_1/2_ < 2 h) was observed in the presence of intestinal enzymes (pancreatin, SIF) at pH = 6.8. This supported the initial hypothesis concerning the expected behavior of the compound in vivo. Further consolidation was provided by observation of very fast turnover in both human and rat hepatocytes and plasma (*T*_1/2_ < 30 min). Indeed, in fresh plasma from rats, ***LaKe*** could not be detected at the first time point, 10 min. The compound also underwent turnover in both rat and human liver microsomes albeit somewhat slower. The data displayed in Figure 5 were performed in the presence of NADPH; however, conversion also occurred in the absence of activating NADPH with 82% (human) and 55% (rat) ***LaKe*** remaining after 60 min. All in all, the results suggest that ***LaKe*** will undergo rapid, enzyme-mediated, hydrolytic turnover in vivo to release its constituents, (*S*)-lactate and (*R*)-1,3-butanediol.

### In Vivo Pharmacokinetics of ***LaKe***

**Figure 6:**
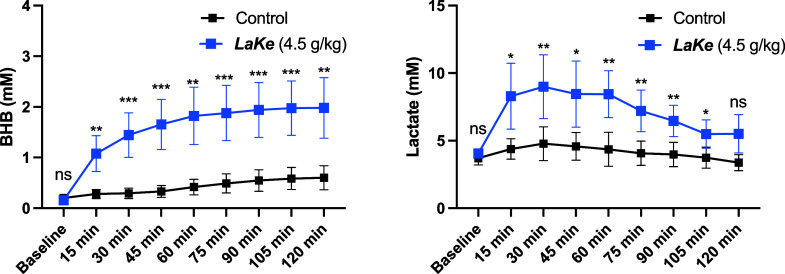
Short-term pharmacokinetics following oral administration of ***LaKe*** in rats. Data are mean ± SD (*N* = 8). ns = nonsignificant; * *P* < 0.05; ** *P* < 0.01; *** *P* < 0.001.

Next, we conducted an experiment involving acute, oral administration of 4.5 g/kg ***LaKe*** to Sprague–Dawley rats and monitored the plasma concentrations of lactate and BHB, respectively, by blood sampling every 15 min until 120 min (Figure 6). To minimize eventual stress-induced changes in metabolite levels, the rats were anesthetized during the experiment and allowed to stabilize for 15 min after instrumentation. During 15 min, following ***LaKe*** dosing, the average plasma concentration of BHB increased from a baseline value of about 0.2–1.1 mM and reached a peak level of 2.0 mM after 105 min. Likewise, plasma levels of lactate also increased rapidly from the baseline (ca. 4 mM) to a peak level of 9.0 mM after 30 min. At 120 min, lactate levels were largely back at the baseline level, whereas BHB levels remained elevated. This experiment clearly demonstrated that oral administration of ***LaKe*** could elevate lactate and BHB levels in vivo. The pharmacokinetic profile observed is compatible with the rapid release of lactate following hydrolysis and slower formation of BHB from oxidation of 1,3-butanediol.

**Figure 7:**
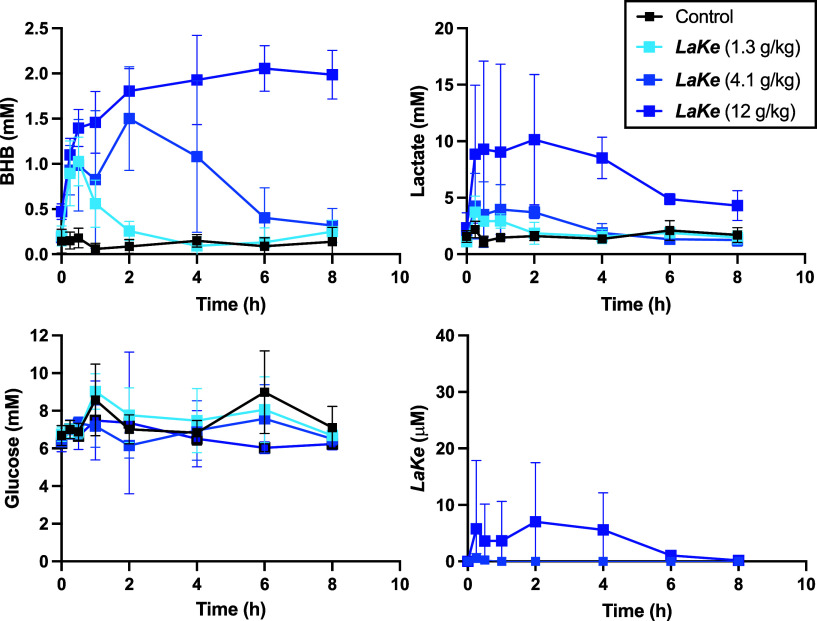
Extended time (8 h) and dose-dependent pharmacokinetics of ***LaKe***. Data are mean ± SD (*N* = 6).

Encouraged by these results, we next conducted an additional experiment in rats to evaluate both an extended pharmacokinetic profile (8 h) and different doses of ***LaKe*** (up to 12 g/kg). Before administration of ***LaKe***, the rats were fasted overnight and maintained on glucose-water to suppress endogenous ketone body and additional lactate formation. In this experiment, the rats were not anesthetized. Again, we observed clear, dose-dependent elevation of plasma levels of both BHB and lactate. The concentrations of BHB remain within a window of 0.5 mM to about 2 mM (corresponding to ca. 5–20 fold increase over baseline) with increasing doses resulting in an extended duration of the induced ketosis (Figure 7). Like the first experiment, lactate levels display a somewhat faster return to baseline values, with peak levels corresponding to approximately 4-fold increase compared to baseline values in the first 2 h at both the low and intermediate doses, the latter with an extended duration (Figure 7). At the high dose, plasma levels of lactate in this experiment reached 10 mM and remained elevated at the final time point (8 h). At the 4.1 g/kg dose, the average area under the curve (AUC) of each metabolite, following subtraction of AUC of vehicle, was similar (lactate: 5233 μM·h, BHB: 5563 μM·h) in accordance with the 1:1 stoichiometry. With respect to blood glucose, we observed a nonsignificant trend toward lower glucose levels with high-dose ***LaKe*** exposure (Figure 7). It should be noted that our study design involved continuous ingestion of 5% glucose water, which per se probably affected these results by increasing insulin levels and decreasing free fatty acid levels (Figure 7). We also assessed the plasma concentrations of ***LaKe*** at the low and high concentration. Encouragingly, even at the maximum dose of 12 g/kg, average plasma levels of ***LaKe*** reached a maximum of just 5 μM. At the lower dose, ***LaKe*** was only detectable at 15 and 30 min with average concentrations of 540 and 260 nM, respectively (Figure 7). At the maximum concentration (12 g/kg) at 2 and 8 h following administration, some of the rats displayed slightly reduced activity and a hunched posture. These observations, however, normalized at 10 h following administration. At the lower doses (1.3 g/kg and 4.1 g/kg), normal behavior was observed at all time points. No signs of toxicity or deviation from normal behavior were observed 10 h following administration for any of the doses. Thus, according to our data, ***LaKe*** appears well tolerated. In accordance with the in vitro assays, the compound is rapidly metabolized and, consequently, results in elevated lactate and BHB levels already after 15 min.

### ***LaKe*** Modulates Plasma Levels of Free Fatty Acids and *N*-L-Lactoyl-phenylalanine

**Figure 8:**
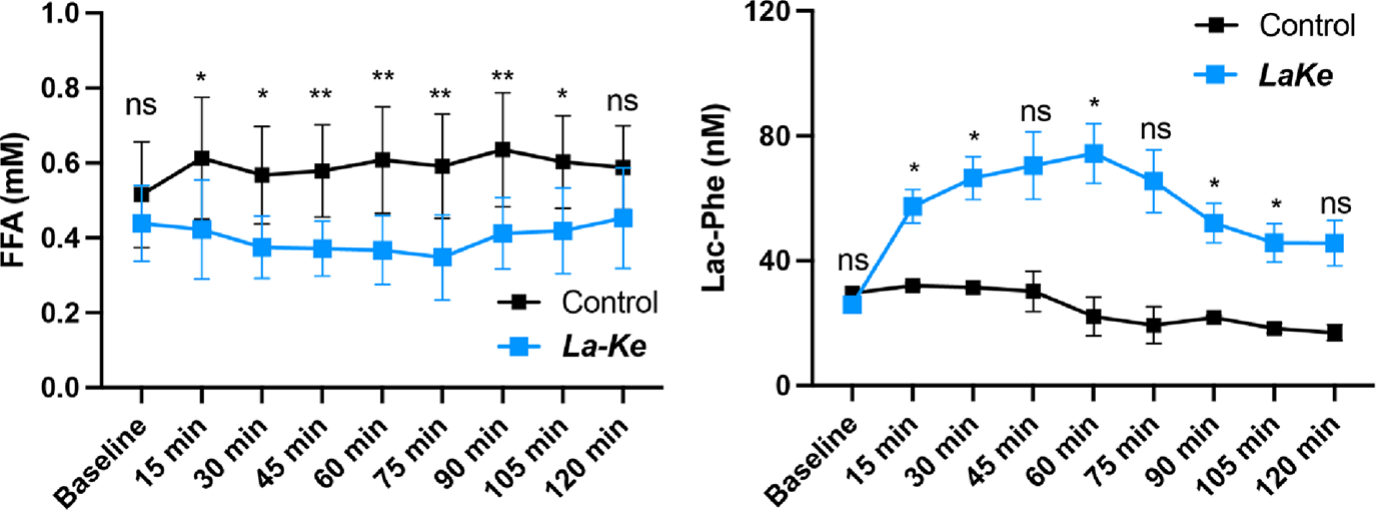
Assessment of plasma levels of free fatty acids (FFA) and *N*-L-lactoyl-phenyalanine (Lac-Phe) following oral administration of ***LaKe*** (4.5 g/kg) in rats. Data are mean ± SD. For FFA (*N* = 8 in each group). For Lac-Phe (*N* = 8 in ***LaKe*** group; *N* = 2 in control group). ns = nonsignificant; * *P* < 0.05; ** *P* < 0.01.

Given that ***LaKe*** allows for the controlled increase of BHB and lactate levels in vivo, ingestion of the compound will naturally impact other biological parameters that either respond to or depend on BHB/lactate levels. Toward this end, we confirmed that ***LaKe*** (4.5 g/kg) afforded a decrease in plasma free fatty acids (FFA)^52,53^ compared to vehicle control from 15 min until 105 min. In this short-term experiment, we observed a trend toward increasing levels of FFA in the vehicle-treated samples compared to baseline (significant at 90 min), which we attribute to fasting of the anesthetized rats, which would naturally lead to lipase activation in adipose tissue and FFA release. On this background, ***LaKe*** still suppressed FFA levels with a maximum effect >40% at the 75 min time point. Furthermore, ***LaKe*** resulted in an increase in the amino acid-derived metabolite *N*-L-lactoyl-phenylalanine (Lac-Phe), which was recently identified to suppress feeding and obesity in rats^16^ and has been found to increase following metformin-treatment of type 2 diabetes in humans (Figure 8).^54^

In this study, we have demonstrated a novel, low-molecular-weight ester that allows efficient supplementation of (*S*)-lactate and (*R*)-beta-hydroxybutyrate following oral administration in rats. The plasma levels obtained mirror those typically observed following fasting (BHB) and during physical activity (lactate), thereby mimicking a subset of the physiological responses triggered by strenuous exercise.^55^ These initial experiments indicate that ***LaKe*** is well-tolerated. We furthermore demonstrate that ***LaKe*** ingestion leads to lower levels of plasma free fatty acids (FFA) compared to the vehicle control and, conversely, to an increase in the appetite-suppressing peptide hormone *N*-L-lactoyl-phenylalanine (Lac-Phe). Overall, these results suggest that ***LaKe*** is an excellent candidate molecule for achieving controlled, systematic elevation of plasma levels of lactate and ketone bodies and may trigger the biological responses associated with these metabolites. Future studies will provide deeper investigations of the cardiometabolic, including broader hormone panels, and cardiovascular and neurological effects of ***LaKe*** supplementation. Further studies into the absolute bioavailability of ***LaKe***-derived (*S*)-lactate and BHB, using tracers, will also be of interest.

## Supporting Information Available

Detailed experimental descriptions related to synthetic chemistry, stability data for ***LaKe*** upon storage, spectroscopic data on final compounds and intermediates, detailed experimental protocols for in vitro and in vivo pharmacokinetics and associated analyses, and copies of NMR spectra (PDF)

## Abbreviations

BDNF: brain-derived neurotrophic factor
BHB: beta-hydroxybutyrate
DIPEA: *N,N*-diisopropylethylamine
FFA: free fatty acids
GLP-1: glucagon-like peptide 1
HCAR1: hydroxycarboxylic acid receptor 1
HCAR2: hydroxycarboxylic acid receptor 2
Lac-Phe: *N*-L-lactoyl-phenylalanine
LEAP2: liver-expressed antimicrobial peptide 2
SGF: simulated gastric fluid.
SIF: simulated intestinal fluid
TBS: *tert*-butyldimethylsilyl
THF: tetrahydrofuran
TBTU: 2-(1*H*-Benzotriazole-1-yl)-1,1,3,3-tetramethylaminium tetrafluoroborate
VEGF: vascular endothelial growth factor
