# Supplementary material for: Preparation and Preclinical Characterization of a Simple Ester for Dual Exogenous Supply of Lactate and Beta-hydroxybutyrate
Source: J Agric Food Chem. 2024 Aug 30;72(36):19883–90. doi: 10.1021/acs.jafc.4c04849 (PMC11403612; doi:10.1021/acs.jafc.4c04849)
Supplement: Supplementary file 1 — jf4c04849_si_001.pdf [file jf4c04849_si_001.pdf]

Supporting Information

Preparation and pre-clinical characterization of a simple ester for dual exogenous supply of lactate and beta-hydroxybutyrate

Rasmus N. Ottosen,[1] Jacob M. Seefeldt,[2] Jakob Hansen,[3] Roni Nielsen,[2] Niels Møller*,[4,5], Mogens Johannsen*,[3], Thomas B. Poulsen*,[1]

[1] Department of Chemistry, Aarhus University, Langelandsgade 140, DK-8000, Aarhus C, Denmark.

[2] Department of Cardiology, Aarhus University Hospital, Palle Juul-Jensens Boulevard 99, DK-8200 Aarhus N, Denmark

[3] Department of Forensic Medicine, Aarhus University, Palle Juul-Jensens Boulevard. 99, DK-8200 Aarhus N, Denmark

[4] Steno Diabetes Center Aarhus, Aarhus University Hospital, Palle Juul-Jensens Boulevard 11, DK-8200 Aarhus N, Denmark Denmark

[5] Department of Clinical Medicine, Aarhus University, Palle Juul-Jensens Boulevard 82, DK-8200 Aarhus N, Denmark Denmark

[*] e-mail: thpou@chem.au.dk, mj@forens.au.dk, niels.moeller@clin.au.dk

Synthetic procedures and characterization data

Synthesis of *LaKe* (3 and 4)

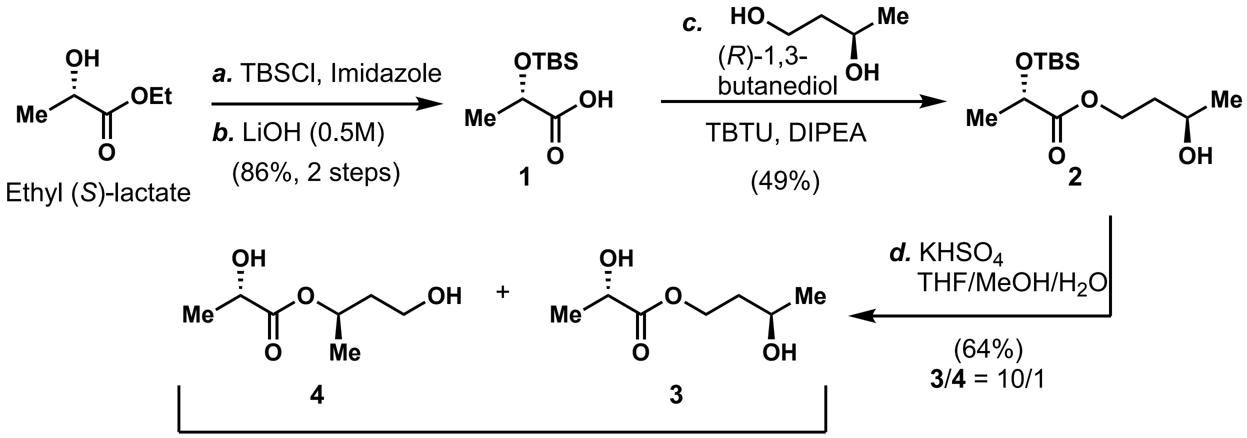

Figure S1

(*S*)-2-((*tert*-butyldimethylsilyl)oxy)propanoic acid (1)

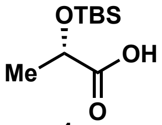

1

To a solution of ethyl (*S*)-(-)-2-hydroxypropionate (14.4 mL, 127 mmol, 1.0 eq.) in anhydrous DMF (250 mL) was added TBSCl (28.7 g, 191 mmol, 1.5 eq.) followed by imidazole (30.3 g, 445 mmol, 3.5 eq.). The mixture was stirred at r.t. for 3 hours before diluted with sat. aq. NaCl and extracted with EtOAc. The organic layers were combined and washed with 5 % aq. HCl and sat. aq. NaCl. The organic layer was then dried over Na_2SO_4, filtered and concentrated to afford ethyl (*S*)-2-((*tert*-butyldimethylsilyl)oxy)propanoate (29.5 g, quant.). Product formation was determined with ^{1}H NMR analysis. No further purification was performed as the product was subjected directly into the following step.

R_f	0.41 (Pentane/EtOAc 98:2, $KMnO_4$).
^{1}H NMR	(400 MHz, $CDCl_3$) δ_H 4.34-4.26 (q, *J* = 6.7 Hz, 1H), 4.24-4.11 (m, 2H), 1.41-1.36 (d, *J* = 6.7 Hz, 3H), 1.30-1.24 (t, *J* = 7.2 Hz, 3H), 0.91-0.87 (s, 9H), 0.11-0.04 (d, *J* = 12.0 Hz, 6H).

To a solution of the (*S*)-2-((*tert*-butyldimethylsilyl)oxy)propanoate (29.5 g, 127 mmol, 1.0 eq.) in THF (400 mL) at 0 °C was added a cooled aq. LiOH solution (508 mL, 0.5 M). The reaction mixture was stirred at r.t. for 5 hours before concentrated to half of the original volume and extracted with Et_2O. The organic extracts were combined before extracted with a sat. aq. $NaHCO_3$ solution. The aqueous layers were combined and acidified with a 1 M aq. $KHSO_4$ solution to reach pH ≈ 3-4. Afterwards, the aqueous solution was extracted thourghly with Et_2O and the organic layers were combined, dried over Na_2SO_4 and concentrated to afford (*S*)-2-((*tert*-

butyldimethylsilyl)oxy)propanoic acid (22.3 g, 86 %) as an oil. Product formation was determined with ^{1}H NMR analysis (^{1}H NMR values are in accordance with reported values)[1].

^{1}H NMR (400 MHz, $CDCl_3$) δ_H 4.38-4.33 (q, J = 6.8 Hz, 1H), 1.46-1.45 (d, J = 6.8 Hz, 3H), 0.94-0.90 (m, 9H), 0.16-0.12 (s, 6H).

(*R*)-3-Hydroxybutyl (*S*)-2-((*tert*)-butdyldimethylsilyl)oxy)propanoate (2)

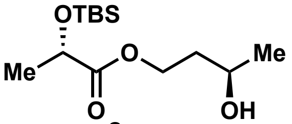

2

(*S*)-2-((t*ert*-butyldimethylsilyl)oxy)propanoic acid (8.42 g, 41.2 mmol, 1.0 eq.), TBTU (19.9 g, 61.8 mmol, 1.5 eq.) and DIPEA (15.1 mL, 86.5 mmol, 2.1 eq.) was dissolved in anhydrous DMF (150 mL) and the mixture was stirred at r.t. for 1 hour. (*R*)-(-)-1,3-butanediol (3.68 mL, 41.2 mmol, 1.0 eq.) in anhydrous DMF (10 mL) was then added and the reaction mixture was stirred o/n. at ambient temperature. The reaction mixture was diluted with CH_2Cl_2 and the resulting mixture was washed with 1 M aq. HCl, aq. $NaHCO_3$ and water sequentially. The organic layers were then combined, dried over Na_2SO_4, filtered and concentrated to afford the crude product. FCC (pentane/EtOAc = 95:5 to 85:15) was performed to afford the pure product (**2**) (5601 mg, 49 %).

R_f 0.38 (Pentane/EtOAc 8:2, $KMnO_4$).
^{1}H NMR (400 MHz, $CDCl_3$) δ_H 4.42-4.30 (m, 2H), 4.21-4.16 (m, 1H), 3.93-3.84 (m, 1H), 2.00-1.97 (d, J = 4.3 Hz, 1H), 1.85-1.69 (m, 2H), 1.42-1.38 (d, J = 6.8 Hz, 3H), 1.24 1.21 (d, J = 6.3 Hz, 3H), 0.91-0.89 (s, 9H), 0.11-0.07 (d, J = 10.2 Hz, 6H).
^{13}C NMR (101 MHz, $CDCl_3$) δ_C 174.6, 68.6, 65.1, 62.3, 38.2, 25.9, 23.6, 21.5, 18.4, -4.8, -5.1.
IR (neat) υ_{max}/cm^{-1} 3440, 2930, 2858, 1737, 1463, 1373, 1256, 1140.
HRMS $[M+Na]^+$ = 299.1649 ; found: 299.1644.
$[\alpha]^{24.0}_D$ -34.6° (C = 10 mg/mL, $CHCl_3$).

(*R*)-3-Hydroxybutyl (*S*)-2-hydroxypropanoate (3) and (*R*)-4-hydroxybutan-2-yl (*S*)-2-hydroxypropanoate (4)

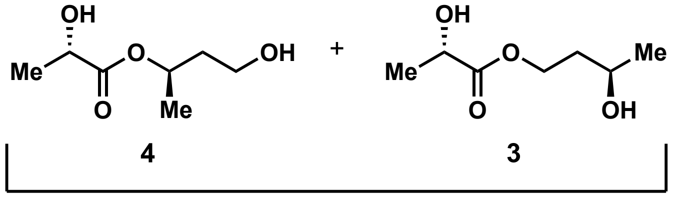

LaKe

To a stirred solution of TBDMS ether (5612 mg, 20.3 mmol, 1.0 eq.) in THF:H_2O:MeOH (2.5:2.5:1, 100 mL) was added $KHSO_4$ (691 mg, 5.08 mmol, 0.25 eq.) and the mixture was stirred at r.t. o/n. MeOH was removed under reduced pressure and water was added before extracted with EtOAc. The organic layers were collected and dried over Na_2SO_4, filtered and concentrated. The crude product was purified with FCC (Pentane/EtOAc = 1:1) to afford the desired products (2100 mg, 68 % ; *r.* **3**:**4** : 10:1).

[1]Mayer, S.C.; Ramanjulu, J; Vera, M.D.; Pfizenmayer, A.J; Joullié, M.M, *J. Org. Chem.*, **1994**, *59*, 5192-5205

R_f 0.19 (Pentane/EtOAc 1:1, CAM).

^{1}H NMR **3:** (400 MHz, $CDCl_3$) δ_H 4.46-4.37 (m, 1H), 4.32-4.23 (m, 2H), 3.95-3.85 (m, 1H), 2.81-2.79 (d, J = 5.3 Hz, 1H), 1.87-1.71 (m, 3H), 1.44-1.39 (d, J = 7.0 Hz, 3H), 1.27-1.22 (d, J = 6.53 Hz, 3H).

4: (400 MHz, $CDCl_3$) δ_H 5.25-5.17 (m, 1H), 4.46-4.37 (m, 1H), 3.74-3.57 (m, 2H), 1.87-1.71 (m, 2H), 1.44-1.39 (d, J = 7.0 Hz, 3H), 1.33-1.30 (d, J = 6.2 Hz, 3H).

^{13}C NMR **3:** (101 MHz, $CDCl_3$) δ_C 176.1, 66.9, 64.9, 63.0, 38.0, 23.8, 20.6.

IR (neat) υ_{max}/cm^{-1} 3380, 2971, 1732, 1457, 1375, 1274, 1209, 1130, 1049.

HRMS $[M+Na]^+$ = 185.0784 ; found: 185.0783.

Chemoenzymatic synthesis of 3 and 4 (*LaKe*):

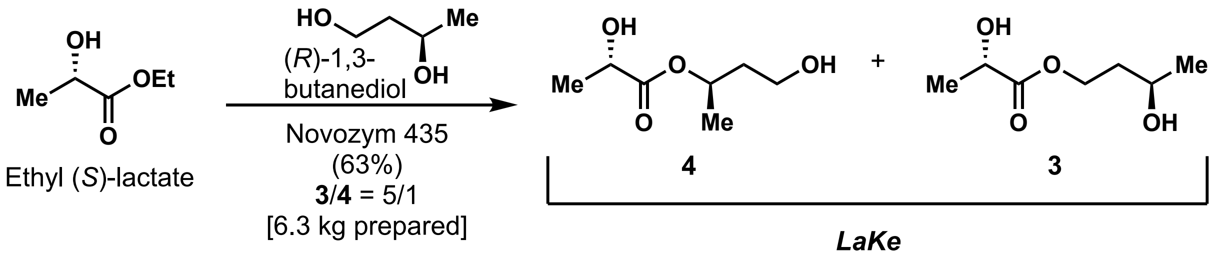

Figure S2

These experiments were carried out at NCK A/S.

Ethyl L-lactate (2.95 kg, 25 mol) and (*R*)-1,3-butanediol (450 g, 5.0 mol) were mixed with gentle mechanical stirring. Novozym 435 (45 g, 0.1 equiv. wt/wt) was added, and the mixture was heated to ca. 35°C under reduced pressure (ca. 25 mbar) to remove any ethanol formed during the reaction. After 28 hours an additional amount of Novozym 435 (22 g) was added, and the mixture was stirred for another 12 hours. In process control at this stage showed a conversion of (*R*)-1,3-butanediol in to ***LaKe*** of 88%. Heating was discontinued, and the reaction was stopped by filtering off the enzyme beads. The reaction mixture was concentrated by distillation using a bath temperature of 65°C and a pressure of 30 to 2-3 mbar. The crude product (821 g) was further purified by high vacuum distillation and suitable fractions were collected at a distillation temperature of l05-110°C and a pressure of ca. 0.1 mbar. The isolated yield was 506 g (62.5%) and the purity was 96.4% (GC-area%).

The same protocol was adopted using the following amounts of starting materials:

Ethyl L-lactate (36.1 kg + 2.2 kg for washing), (*R*)-1,3-butanediol (5.5 kg), Novozym 435 (0.50 kg + 0.25 kg) to afford ***LaKe*** (6.3 kg, 63%) with a purity of 97.1% (GC-area%). Impurities were 1,3-butanediol (1.3%) and the bis-lactate ester (1.1%)

The sample was stored for 24 months under different conditions and re-analyzed by GC-FID with the following results.

Table S1 – Stability of LaKe (neat) under different conditions

Entry	Condition	LaKe purity	1,3-butanediol	Bis-lactate ester
1	**t = 0**	**97.1%**	**1.3%**	**1.1%**
2	**t = 24 months @ -15 °C**	**96.6%**	**1.7%**	**1.1%**
3	**t = 24 months @ 5 °C**	**96.6%**	**1.7%**	**1.1%**
4	**t = 24 months @ 20 °C**	**95.9%**	**1.8%**	**1.3%**

GC-FID. Procedure. Column: CP-SIL 5 CB, 25 m x 0.53 mm, 5.0 μm, Part No. CP7675; Carrier gas: Helium; Mode: Constant flow. Oven. Initial temp: 60 °C; Initial time: 1 min; Rate: 6 °C/min; Final temp: 265 °C; Final time: 5 min. Inlet. Mode: Split; Temp: 120 °C; Flow: 1.9 ml/min; Gas saver: Off. Detector. Temp: 265 oC; H_2 flow: 40 mL/min; Air flow: 450 mL/min; Mode: Constant make up; Mkup: 30 mL/min; Integration time: 40 min; Injection vol.: 2 μL.

Synthesis of D-lactic acid ester 7 and 8 (*D-LaKe*):

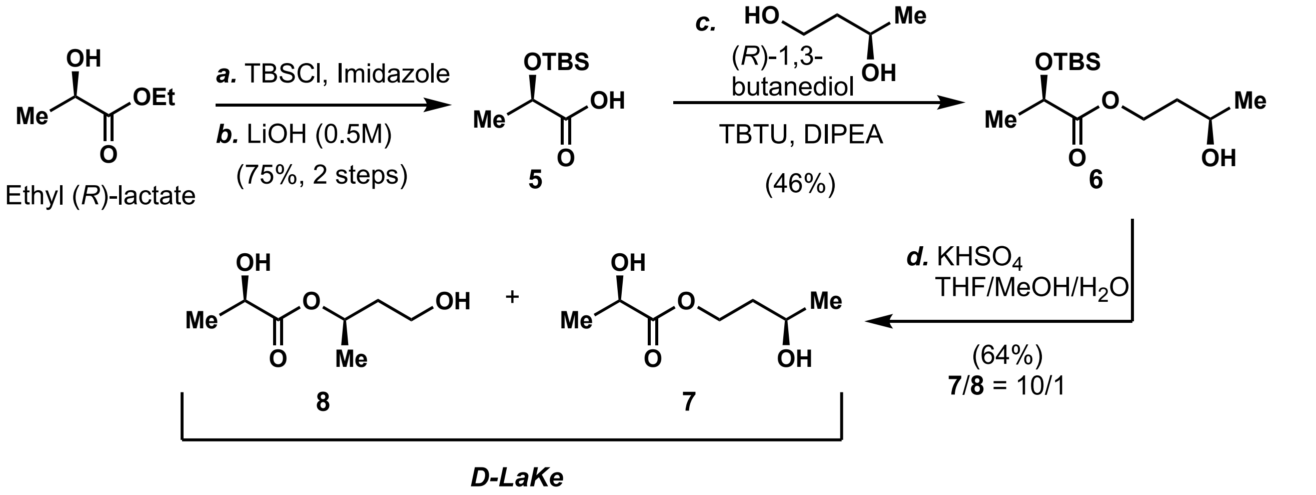

Figure S3

(*R*)-2-((*tert*-butyldimethylsilyl)oxy)propanoic acid (5)

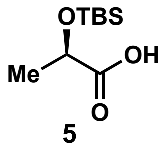

To a solution of ethyl (*R*)-2-hydroxypropionate (6.74 mL, 59.3 mmol, 1.0 eq.) in anhydrous DMF (100 mL) was added TBSCl (13.4 g, 89 mmol, 1.5 eq.) followed by imidazole (14.1 g, 208 mmol, 3.5 eq.). The mixture was stirred at r.t. for 3 hours before diluted with sat. aq. NaCl and extracted with EtOAc. The organic layers were combined and washed with 5 % aq. HCl and sat. aq. NaCl. The organic layer was then dried over Na_2SO_4, filtered and concentrated to afford ethyl (*R*)-2-(*tert*-butyldimethylsilyloxy)propanoate (13.7 g, quant.). Product formation was determined with ^{1}H NMR analysis. No further purification was performed as the product was subjected directly into the following step.

R*f* 0.41 (Pentane/EtOAc 98:2, $KMnO_4$).

^{1}H NMR (400 MHz, $CDCl_3$) δ_H 4.34-4.26 (q, *J* = 6.7 Hz, 1H), 4.24-4.11 (m, 2H), 1.41-1.36 (d, *J* = 6.7 Hz, 3H), 1.30-1.24 (t, *J* = 7.16 Hz, 3H), 0.91-0.87 (s, 9H), 0.11-0.04 (d, *J* = 12.0 Hz, 6H).

To a solution of the (*R*)-2-(*tert*-butyldimethylsilyloxy)propanoate (13.7 g, 59.3 mmol, 1.0 eq.) in THF (400 mL) at 0 °C was added a cooled aq. LiOH solution (400 mL, 0.3 M). The reaction mixture was stirred at r.t. for 5 hours before concentrated to half of the original volume and extracted with Et_2O. The organic extracts were combined before extracted with a sat. aq. $NaHCO_3$ solution. The aqueous layers were combined and acidified with a 1 M aq. $KHSO_4$ solution to reach pH ≈ 3-4. Afterwards, the aqueous solution was extracted thourghly with Et_2O and the organic layers were combined, dried over Na_2SO_4 and concentrated to afford (*R*)-2-(t*ert*-butyldimethylsilyloxy)propanoic acid (9085 mg, 75 %) as an oil. Product formation was determined with ^{1}H NMR analysis (^{1}H NMR values are in accordance with reported values)[2].

^{1}H NMR (400 MHz, $CDCl_3$) δ_H 4.38-4.33 (q, *J* = 6.9 Hz, 1H), 1.46-1.45 (d, *J* = 6.9 Hz, 3H), 0.94-0.90 (m, 9H), 0.16-0.12 (s, 6H).

(*R*)-3-Hydroxybutyl (*S*)-2-((*tert*)-butdyldimethylsilyl)oxy)propanoate (6)

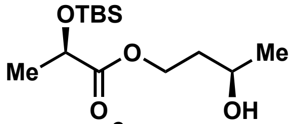

6

(*R*)-2-((*tert*-butyldimethylsilyl)oxy)-propanoic acid (8.99 g, 44.0 mmol, 1.0 eq.), TBTU (21.3 g, 66.0 mmol, 1.5 eq.) and DIPEA (16.1 mL, 92.4 mmol, 2.1 eq.) was dissolved in anhydrous DMF (175 mL) and the mixture was stirred at r.t. for 1 hour. (*R*)-(-)-1,3-butanediol (3.93 mL, 44.0 mmol, 1.0 eq.) in anhydrous DMF (10 mL) was then added and the reaction mixture was stirred o/n. at ambient temperature. The reaction mixture was diluted with CH_2Cl_2 and the resulting mixture was washed with 1 M aq. HCl, aq. $NaHCO_3$ and water sequentially. The organic layers were then combined, dried over Na_2SO_4, filtered and concentrated to afford the crude product. FCC (pentane/EtOAc = 95:5 to 85:15) was performed to afford the pure product (5576 mg, 46 %).

R_f 0.38 (Pentane/EtOAc 8:2, $KMnO_4$).
^{1}H NMR (400 MHz, $CDCl_3$) δ_H 4.44-4.36 (m, 1H), 4.36-4.29 (q, *J* = 6.6 Hz, 1H), 4.20-4.13 (m, 1H), 3.93-3.82 (m, 1H), 2.01-1.96 (d, *J* = 4.4 Hz, 1H), 1.86-1.67 (m, 2H), 1.42-1.38 (d, *J* = 6.7 Hz, 3H), 1.25-1.20 (d, *J* = 6.2 Hz, 3H), 0.93-0.87 (s, 9H), 0.11-0.07 (d, *J* = 10.4 Hz, 6H).
IR (neat) υ_{max}/cm^{-1} 3449, 2958, 2930, 2900, 2858, 1736, 1253, 1138.
HRMS $[M+Na]^+$ = 299.1649 ; found: 299.1658.
$[\alpha]^{24.0}_D$ +17.1° (C = 8.5 mg/mL, $CHCl_3$).

[2] Taiwan Liposome Co Ltd.; TLC Biopharmaceuticals, INC. – US2010/227877, 2010, A1, Page Column 5.

(*R*)-3-Hydroxybutyl (*R*)-2-hydroxypropanoate (7) and (*R*)-4-hydroxybutan-2-yl (*R*)-2-hydroxypropanoate (8)

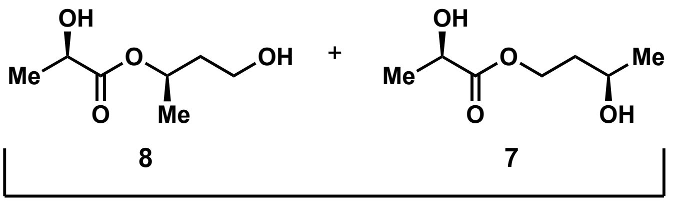

D-LaKe

To a stirred solution of TBDMS ether (5576 mg, 20.2 mmol, 1.0 eq.) in THF:H_2O:MeOH (2.5:2.5:1, 100 mL) was added $KHSO_4$ (668 mg, 5.05 mmol, 0.25 eq.) and the mixture was stirred at r.t. o/n. MeOH was removed under reduced pressure and water was added before extracted with EtOAc. The organic layers were collected and dried over Na_2SO_4, filtered and concentrated. The crude product was purified with FCC (Pentane/EtOAc = 1:1) to afford the desired products (2110 mg, 64 %, *r*. **7**:**8** : 10:1).

R_f	0.19 (Pentane/EtOAc 1:1, CAM).
^{1}H NMR	**7:** (400 MHz, $CDCl_3$) δ_H 4.49-4.38 (m, 1H), 4.33-4.20 (m, 2H), 3.95-3.83 (m, 1H), 2.82-2.74 (d, *J* = 5.0 Hz, 1H), 1.98-1.61 (m, 3H), 1.44-1.39 (d, *J* = 6.9 Hz, 3H), 1.27 1.22 (d, *J* = 6.2 Hz, 3H).
	**8:** (400 MHz, $CDCl_3$) δ_H 5.25-5.17 (m, 1H), 4.49-4.38 (m, 1H), 3.71-3.64 (m, 1H), 3.63-3.54 (m, 1H), 1.98-1.61 (m, 3H), 1.44-1.39 (d, *J* = 6.9 Hz, 3H), 1.34-1.20 (d, *J* = 6.2 Hz, 3H).
^{13}C NMR	**7:** (101 MHz, $CDCl_3$) δ_C 176.1, 66.9, 64.9, 63.0, 37.9, 23.7, 20.5.
IR (neat)	υ_{max}/cm^{-1} 3377, 2971, 2901, 1732, 1376, 1209, 1128, 1079, 1049.
HRMS	$[M+Na]^+$ = 185.0784 ; found: 185.0790.

In vitro stability of _LaKe_ – Detailed protocol

Stability of LaKe in simulated gastric fluid (SIF)

Preparation of SGF-solution: 0.04 g NaCl and 0.064 g pepsin was dissolved H_2O and aqueous HCl was used to adjust pH (total volume of solution 20 mL). The pH of the test solution was about 1.20 ± 0.05.

Procedure: 2 µL of a 10 mg/ml working solution of ***LaKe*** in DMSO was spiked into 96-deep-well plates corresponding to timepoints T0, T60, T120, T360 and T1440. Experiments were performed in duplicate. 198 µL of SGF solution was then transferred to the individual wells (except T0) to reach 0.1 mg/ml as the final test concentration for each time point (60, 120, 360, 1440 minutes). The final concentration of DMSO in the incubation mixture was 1%. Samples were incubated at 37°C, 600 rpm for the appointed time. At the chosen time points (60, 120, 360, 1440 minutes) samples were immediately mixed with 400 µL of cold acetonitrile containing 200 ng/mL tolbutamide and labetalol (internal standards). 200 µL of suspension was removed and mixed completely with 400 µL of cold acetonitrile containing 200 ng/mL tolbutamide and labetalol again. The T0 samples were prepared by transferring 198 µL of SGF solution to corresponding well after adding 400 µL of cold acetonitrile containing 200 ng/mL tolbutamide and then mixing completely. The samples were

centrifuged at 4000 rpm, 4℃ for 20 min. 60 µL of supernatant was mixed with 180 µL of purified water for subsequent LC/MS/MS analysis.

LC-MS/MS Condition: ACQUITY UPLC BEH C18 1.7um 2.1 * 50mm Column (Part No.186002350). Mobile phase: A: 0.1% Formic Acid in Water; B: 0.1% Formic Acid in ACN.

Stability of LaKe in simulated intestinal fluid (SIF)

Preparation of SIF-solution: 0.136 g KH_2PO_4 and 0.064 g pancreatin was dissolved H_2O to a total volume of solution 20 mL. The pH of the test solution was adjusted to 6.80 ± 0.05.

Procedure: 2 µL of a 10mg/ml working solution of LaKe in DMSO was spiked into 96-deep-well plates corresponding to timepoints T0, T60, T120, T360 and T1440. Experiments were performed in duplicate. 198 µL of SIF solution was then transferred to the individual wells (except T0) to reach 0.1mg/ml as the final test concentration for each time point (60, 120, 360, 1440 minutes). The final concentration of DMSO in the incubation mixture was 1%. Samples were incubated at 37 ℃, 600 rpm for the appointed time. At the chosen time points (60, 120, 360, 1440 minutes) samples were immediately mixed with 400 µL of cold acetonitrile containing 200 ng/mL tolbutamide and labetalol (internal standards). 200 µL of suspension was removed and mixed completely with 400 µL of cold acetonitrile containing 200 ng/mL tolbutamide and labetalol again. The T0 samples were prepared by transferring 198 µL of SIF solution to corresponding well after adding 400 µL of cold acetonitrile containing 200 ng/mL tolbutamide and then mixing completely. The samples were centrifuged at 4000 rpm, 4℃ for 20 min. 60 µL of supernatant was mixed with 180 µL of purified water for subsequent LC/MS/MS analysis.

LC-MS/MS Condition: ACQUITY UPLC BEH C18 1.7um 2.1 * 50mm Column (Part No.186002350). Mobile phase: A: 0.1% Formic Acid in Water; B: 0.1% Formic Acid in ACN.

Stability of LaKe in plasma

Frozen human plasma was thawed in a water bath at 37 ℃ prior to the experiment. The plasma was centrifuged at 4000 rpm for 5 min and clots, if any, were removed. Using an Apricot automation workstation, 98 µL/well of plasma (rat or human) was added to all 96-well reaction plates. (Blank, T0, T10, T30, T60 and T120). An Apricot automation workstation was used to add 2 µL/well of working solution (100 µM) to all reaction plates except the blank (T0, T10, T30, T60 and T120). All reaction plates containing mixtures of compound and plasma were incubated at 37°C in water bath. At the end of incubation, 500 µL of stop solution (200 ng/mL tolbutamide and 200 ng/mL labetalol in ACN) was added and mixed thoroughly to precipitate protein. Each plate was sealed and shaken for 20 minutes. After shaking, each plate was centrifuged at 4000 rpm and 4°C for 20 minutes. After centrifugation, an Apricot automation workstation was used to transfer 150 µL of supernatant from each reaction plate to its corresponding bioanaylsis plate. Each bioanalysis plate was sealed and shaken for 10 minutes prior to LC-MS/MS analysis.

LC-MS/MS Condition: ACQUITY UPLC HSS T3 1.8um 2.1 * 50mm Column (Part No. 186003538). Mobile phase: A: 0.1% Formic Acid in Water; B: 0.1% Formic Acid in ACN.

Hepatocyte stability of *LaKe*

Cells: Rat hepatocytes (BioreclamationIVT, M0005); Human hepatocytes (BioreclamationIVT, X008001)

Thawing Medium: Williams' Medium E containing 5% fetal bovine serum and 30% Percoll solution and other supplements.

Incubation Medium: Williams' Medium E (no phenol red) containing 2 mM L-Glutamine and 25 mM HEPES.

Stop Solution: Acetonitrile containing 200 ng/mL tolbutamide and labetalol as internal standards.

Dilution Solution: Ultra-pure water.

Preparation of Quenching Plate: Transfer 125 µL of stop solution (acetonitrile containing 200 ng/mL tolbutamide and 200 ng/mL labetalol as internal standards) in a set of pre-labeled 96-well plates.

Cryopreserved cells were thawed, isolated and suspended in Incubation Medium, then diluted with pre-warmed Incubation Medium to 0.5×10^6 cells/mL. 198 µL of pre-warmed cell suspensions were added in 96-well plates. 2 µL dosing solution (10 mg/mL stock) were added to each well of 96-well plate in duplicate. For T0 Samples, mixing to achieve a homogenous suspension was performed for about 1 min, then then 25 µL of each sample was immediately transferred into wells containing 125 µL of ice-cold stop solution followed by mixing. All plates were incubated at 37°C in a 95% humidified incubator at 5% CO_2 with constant shaking at about 600 rpm. At 15, 30, 60 and 90 min, the samples were mixed and then 25 µL of each sample at each time point was transferred to a well containing 125 µL of ice-cold stop solution followed by mixing. Medium Control (MC) sample plates are prepared at T0 and T90 by adding the same components to each well except cell suspensions. The plates were vortexed immediately on a plate shaker at 500 rpm for 10 minutes. Then, all sample plates were centrifuged at 3220 x g for 20 min at 4°C. After centrifugation, 80 µL/well of supernatant in the sample plates were transferred to another set of 96-well plates containing 240 µL of ultra-pure water. Analytical plates were sealed and stored at 4°C until LC-MS/MS analysis.

Microsomal stability of *LaKe*

Microsomes: Human (Cat No. 452117, Corning, Lot No. 38297); SD Rat (Cat No. R1000, Xenotech, Lot No. 2110178)

NADPH: β-Nicotinamide adenine dinucleotide phosphate reduced form, tetrasodium salt; NADPH·4Na (Vendor: BONTAC, Cat. No. BT04). The appropriate amount of NADPH powder was weighed and diluted into a 10 mM $MgCl_2$ solution (working solution concentration: 10 mM; final concentration in reaction system: 1 mM)

Quenching solution: Cold (4°C) acetonitrile (ACN) containing 200 ng/mL tolbutamide and 200 ng/mL labetalol as internal standards (IS)

Liver microsomes were diluted to 0.56 mg/mL (Human) and 0.189 mg/ml (SD Rat) in 100 mM phosphate buffer. 445 µL microsome working solutions (0.56 mg/mL & 0.189 mg/mL) were transferred into pre-warmed 'Incubation' plates (T60 and NCF60) and incubation was continued for 10 min at 37°C with constant shaking.

54 µL liver microsomes were transferred to blank plates and then 6 µL NAPDH-solution was added, and then 180 µL quenching solution was added.

5 µL compound working solution (100 µM) was added into 'incubation' plates (T60 and NCF60) containing microsomes and mixed thoroughly. For the NCF60 plate, 50 µL of 100 mM phosphate buffer was added and mixed thoroughly. The plate was incubated at 37°C for 60 min while shaking. In 'Quenching' plate T0, 180 µL quenching solution and 6 µL NAPDH cofactor solution was added. The plate was chilled to prevent evaporation. For the T60 plate, the contents were mixed thoroughly and immediately 54 µL of the mixture (for the 0-min time point) was transferred to the 'Quenching' plate. Then 44 µL NAPDH cofactor solution was added to incubation plate and the timer was started. The plate was incubated at 37°C for 60 min while shaking. At 5, 15, 30, 45, and 60 min, 180 µL quenching solution was added to 'Quenching' plates, mixed once, and 60 µL sample from T60 plate per time point was transferred to 'Quenching' plates. All sampling plates were shaken for 10 min, then centrifuged at 4000 rpm for 20 minutes at 4°C. 80 µL of supernatant was transferred into 240 µL ultrapure water, and mixed by plate shaker for 10 min. Each plate was sealed and shaken for 10 minutes prior to LC-MS/MS analysis.

Short term pharmacokinetics of *LaKe* in rats – Detailed protocols for LC-MS/MS and FFA analyses

BHB quantification by LC-MS/MS

Forty µL rat plasma was diluted with 160 µL water in a 2 mL tube, added 100µL stable isotope labelled internal standard solution (BHB-13C4 at 480µM (Cambridge Isotope Laboratories cat# CLM-3853-PK) and L-lactic acid-3-3-3-d3 at 870µM (Sigma cat#616567)) and 100 µL methanol (Merck hypergrade) followed by vortex mixing. The sample was added 600 µL acetonitrile (Merck hypergrade), vortex mixed, incubated for 5 min at room temperature, and then centrifuged at 10,000×g for 5 min. The supernatant was transferred to a new 2 mL tube, and designated extract A.

The extract was purified through two consecutive solid phase extraction (SPE) procedures as described previously.[3]

Briefly, a 600 µL aliquot of extract A was mixed with 250 µL of water and incubated for 5 min, and then passed through a Strata-X-C (SPE) cartridge (3 mL with 60 mg polymeric strong cation exchange (SCX) sorbent, Phenomenex) that were preconditioned with 1 mL of methanol followed by 1 mL of water. The eluate was collected, and the cartridge was washed with 850 µL of 60% acetonitrile, which was collected in the same tube. A volume of 850 µL of the combined eluate was passed through a SAX SPE column (cartridge contained 100 mg of a strong anion exchange sorbent, Isolute Biotage), which was preconditioned with 1 mL of methanol, 1 mL of 1 M ammonium acetate and 1 mL of water. The cartridge was washed with 1 mL of 80% acetonitrile and the substances were eluted from the column by 2 mL of 1% formic acid in acetonitrile. The eluate was evaporated at 30 °C under a stream of nitrogen and then redissolved in 200 µL 0.1% acetic acid in acetonitrile.

[3] Sørensen, L. K. *et al*. Simultaneous Determination of β-Hydroxybutyrate and β-Hydroxy-β-methylbutyrate in Human Whole Blood Using Hydrophilic Interaction Liquid Chromatography Electrospray Tandem Mass Spectrometry. *Clin. Biochem*. **2013**, *46*, 1877-1883. https://doi.org/10.1016/j.clinbiochem.2013.08.011

Pure calibrant samples equivalent with BHB concentrations in the original samples of 5, 50, 250, 500, 750, and 1000 µM were prepared by mixing standard solutions in methanol of BHB reference compound (DL-β-Hydroxybutyric acid sodium salt, Sigma Cat#H6501) with stable isotope labelled internal standard solution. After evaporation of the solvent, the residue was redissolved in 400 µL of 0.1% acetic acid in acetonitrile.

A 10 µL sample volume was analysed by hydrophilic interaction (LC–MS/MS) with an ultra high performance liquid chromatography system (Waters Acquity UPLC) coupled to a triple–quadrupole mass spectrometer (Waters Xevo TQ MS). The column was a SeQuant ZIC HILIC column (5 µm, 200 Å, 2.1 mm × 100 mm, Merck) maintained at 30 °C and the column flow rate was 0.2 mL/min running. The initial mobile phase flow was set at 5% mobile phase A (1 mM ammonium acetate) and 95% mobile phase B (acetonitrile). The mobile phase was changed through a linear gradient to 45% A over 4 min and then to 95% A over 0.2 min. After 6 minutes, the gradient was returned to 5% A over 0.5 min, and the column was equilibrated for 4.5 min giving a total runtime of 11 min.

The mass spectrometer was operated in the negative ion mode with a capillary voltage of -2.5 kV and the source and desolvation temperatures were set at 150 °C and 600 °C, while the cone and desolvation nitrogen gas flows were set at 50 L/h and 800 L/h. Multiple reaction monitoring was used for detection of the analytes with the following transitions (m/z) and cone voltage/collision energies (CV/CE), BHB (m/z: 103→59, CV/CE: 18V/10eV) and BHB-13C4 (m/z: 107→61, CV/CE: 18V/10eV). The data acquisition and processing were performed using MassLynx 4.1 (Waters). Calibration curves were created from weighted (1/x) linear regression analysis of the IS-normalised peak areas of the calibrant samples (analyte area/IS area). BHB concentrations in rat plasma samples were derived from their peak area ratio with reference to the calibration curve and taking the plasma sample dilution into account.

Lactate quantification by LC-MS/MS.

Aliquots of 100µL of extract A (prepared as described for BHB analysis) and matched pure calibrator samples at the following lactate concentration levels (5, 50, 250, 500, 750, 1000, or 2000 µM) were transferred into a 1mL 96-deep-well plate (Eppendorf) and evaporated to dryness under a nitrogen flow at 30 °C. The samples were re-constituted in 300 µL water with 0.2% formic acid.

A 7.5 µL sample volume was injected to an ultra high performance liquid chromatography system (Waters Acquity UPLC) coupled to a triple–quadrupole mass spectrometer (Waters Xevo TQS) with a C18 column (Waters Acquity UPLC HSS-C18, 1.8µm, 100x2.1 mm) maintained at 40 °C. The flow rate was 0.4 mL/min and the initial condition was 98% mobile phase A (water with 0.2% formic acid and 2% mobile phase B (methanol with 0.1% formic acid) that was changed through a linear gradient to 20% B over 3 min. and then changed to 90% B at 3.5 min. After 4 min., the gradient was returned to 98% A and maintained there for 2 min, giving a total run time of 6 min.

The sample was introduced to the mass spectrometer through electrospray ionization in the negative mode with a capillary voltage of -2.2 kV and the source and desolvation temperatures were 150 and 500 °C and the nitrogen gas flows were 50 L/h (cone) and 800 L/h (desolvation). The analytes were detected in the multiple reaction monitoring mode with the following transitions (m/z) and cone voltage/collision energies (CV/CE),

lactate (m/z: 89→43.1, CV/CE: 40V/14eV) and lactate-D3 (m/z: 91.7→45.1, CV/CE: 40V/14eV). The data acquisition and processing were performed using MassLynx 4.2 (Waters).

Calibration curves were constructed by linear regression of the peak area ratio of the pure calibrant samples (analyte/internal standard) versus the nominal analyte concentrations with a weighting factor of 1/x and based on eight points (including the blank). Lactate concentrations of rat plasma samples were derived from their peak area ratio with reference to the calibration curve and taking the plasma sample dilution into account.

<u>FFA-quantification</u>

Concentrations of non-esterified free fatty acids (FFA) were measured using a NEFA-HR(2) kit (Wako, Chemicals Gmbh, Germany). Absorbance was measured by spectrometry (PHERAstar FS, BMG LABTECH, Ortenberg, Germany).

<u>Quantification of *N*-L-lactoyl phenylalanine (Lac-Phe) by LC-MS/MS</u>

LC-MS/MS analyses were carried out as follows: Forty µL rat plasma was diluted with 100 µL water in a 2 mL tube, added 115 µL methanol (Merck hypergrade) and 420 µL acetonitrile (Merck hypergrade), vortex mixed, incubated for 5 min at room temperature, and then centrifuged at 10,000 ×g for 5 min. Five hundred microliter supernatant was transferred to a 96-well plate (Eppendorf 1 mL deepwell), evaporated to dryness under a nitrogen gas flow at 30 °C, and then reconstituted in 100 µL water with 0.1 % formic acid.

Pure calibrant samples were prepared from an authentic standard compound (*N*-L-lactoyl phenylalanine), using the plasma sample preparation method at concentrations equivalent to 0, 1, 5, 10, 50, 100, 500, and 1000 µM in the original sample. The lower limit of quantification for *N*-L-lactoyl phenylalanine (Lac-Phe) quantification was estimated to 10 nM.

A 7 µL sample volume was injected to an ultra high performance liquid chromatography system (Waters Acquity UPLC) coupled to a triple–quadrupole mass spectrometer (Waters Xevo TQS) with a T3-UPLC column (Waters Acquity UPLC BEH T3, 1.8µm, 100x2.1 mm) maintained at 40 °C. The flow rate was 0.35 mL/min and the separation was initiated with 100% mobile phase A (water with 0.1% formic acid) for 2 min, and then changed through linear gradients to 40% B (methanol:acetonitrile 1;1 with 0.1 % formic acid) (2-6 min), to 60% B (6-7) min, and to 88% B (7-8 min) and to 100% B (9-10 min). A linear gradient back to 100% A at 11 min was maintained for 3 minutes for column equilibration resulting in a total runtime of 14 minutes.

The sample was introduced to the mass spectrometer through electrospray ionization in the negative mode with a capillary voltage of 2.2 kV and the source and desolvation temperatures were 150 and 500 °C, and the nitrogen gas flows were 50 L/h (cone) and 800 L/h (desolvation). The analytes were detected in the multiple reaction monitoring mode with the following transitions (m/z) and cone voltage/collision energies (CV/CE), L-Lac-Phe (quantification ion m/z: 236.2→88.1, CV/CE: 25V/15eV and qualifier ion m/z: 236.2→147.1, CV/CE: 25V/15eV). The data acquisition and processing including peak integration were performed using MassLynx 4.2 (Waters).

Calibration curves were constructed by linear regression of the peak area of the pure calibrant samples versus the nominal analyte concentrations and based on eight points. The Lac-Phe concentrations in rat plasma samples were derived from their peak area with reference to the calibration curve.

Extended pharmacokinetics of *LaKe* in rats – Detailed protocols for LC-MS/MS analyses

3-hydroxybutyl 2-hydroxypropanoate (***LaKe***):

System: Sciex Triple Quad 6500 Plus; Column: ACQUITY UPLC HSS T3 1.8 µm 2.1 × 50 mm Column

Column temperature: 50.0 C; Flow rate: 0.6 mL/min

Mobile Phase:

Mobile Phase A: 0.1% HCOOH & 2mM $HCOONH_4$ in water/ACN (v:v, 95:5)

Mobile Phase B: 0.1% HCOOH & 2mM $HCOONH_4$ in ACN/water (v:v, 95:5)

Table S2 - Gradient

Time (min)	Mobile Phase B (%)
Initial	0
0.2	0
1.2	100
1.4	100
1.41	0
1.6	0

3-hydroxybutyl 2-hydroxypropanoate (***LaKe***) was detected by SRM. ESI: positive; $[M\text{-}H2O+H]^+$m/z: 145.20/55.20 Da; retention time: 0.724 min

Lactate:

System: Sciex Triple Quad 6500 Plus; Column: ACQUITY UPLC BEH Amide 1.7 µm 2.1 × 100 mm Column

Column temperature: 40.0 C; Flow rate:0.6 mL/min

Mobile Phase:

Mobile Phase A: 0.2% NH_3-H20 and 10mM NH_4OAc in water:ACN(95:5,v/v)

Mobile Phase B: 0.2% NH_3-H20 and 10mM NH4OAc in water:ACN(5:95,v/v)

Table S3 - Gradient

Time (min)	Mobile Phase B (%)
Initial	98
0,6	98
2,2	65
2,8	65
2,81	98
4,5	98

Lactate was detected by SRM; ESI: Negative; $[M-H]^-$ m/z: 89.00/43.10 Da; retention time: 2.1 min.

Beta-hydroxybutyrate:

System: Sciex Triple Quad 6500 Plus; Column: ACQUITY UPLC HSS T3 1.8 µm 2.1 × 150 mm Column;

Column temperature: 50.0 C; Flow rate:0.4 mL/min

Mobile Phase:

Mobile Phase A: 0.1% HCOOH in water

Mobile Phase B: 0.1% HCOOH in water:ACN(50:50,v/v)

Table S4 - Gradient

Time (min)	Mobile Phase B (%)
Initial	3
0.8	3
4.2	7
6.7	95
7.4	95
7.41	3
8	3

Beta-hydroxybutyrate was detected by SRM; ESI: Negative; [M-H]-m/z: 103.00/59.00 Da; retention time: 2.14 min.

NMR Spectra

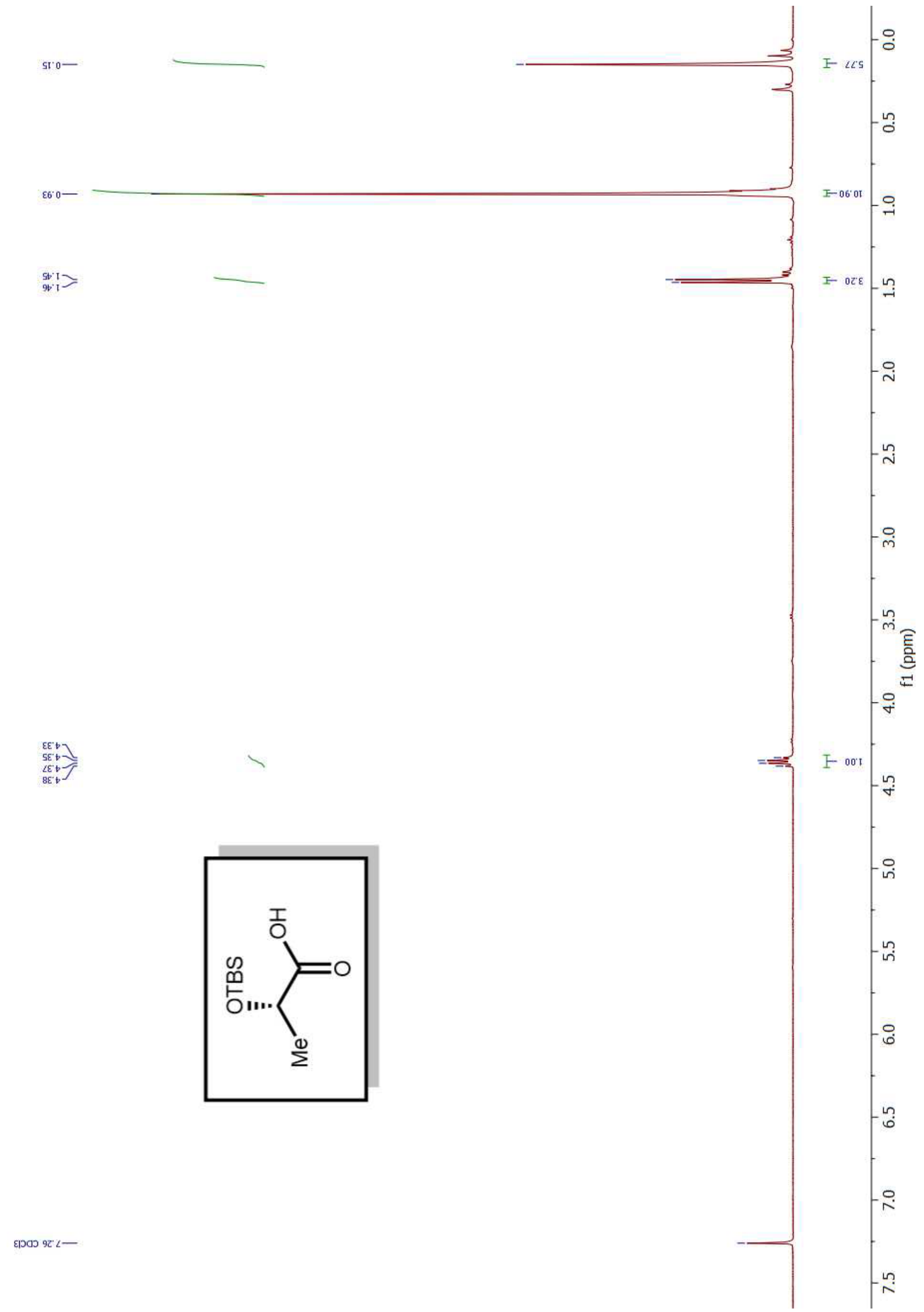
0.15
0.93
1.45
1.46
4.33
4.35
4.37
4.38
7.26 CDCl3
5.77
10.90
3.20
1.00
OTBS
OH
O
Me
f1 (ppm)

Figure S4

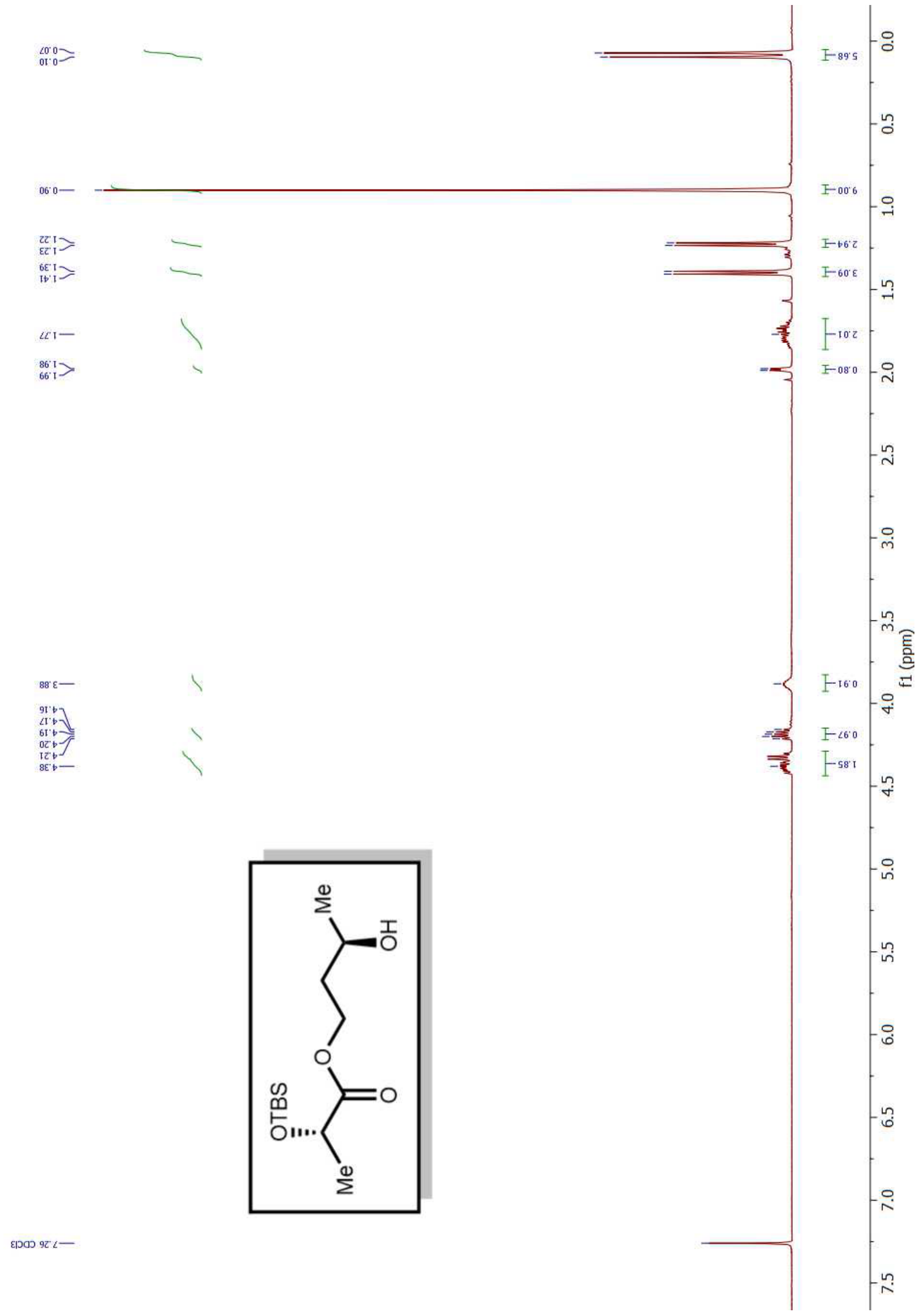

Figure S5

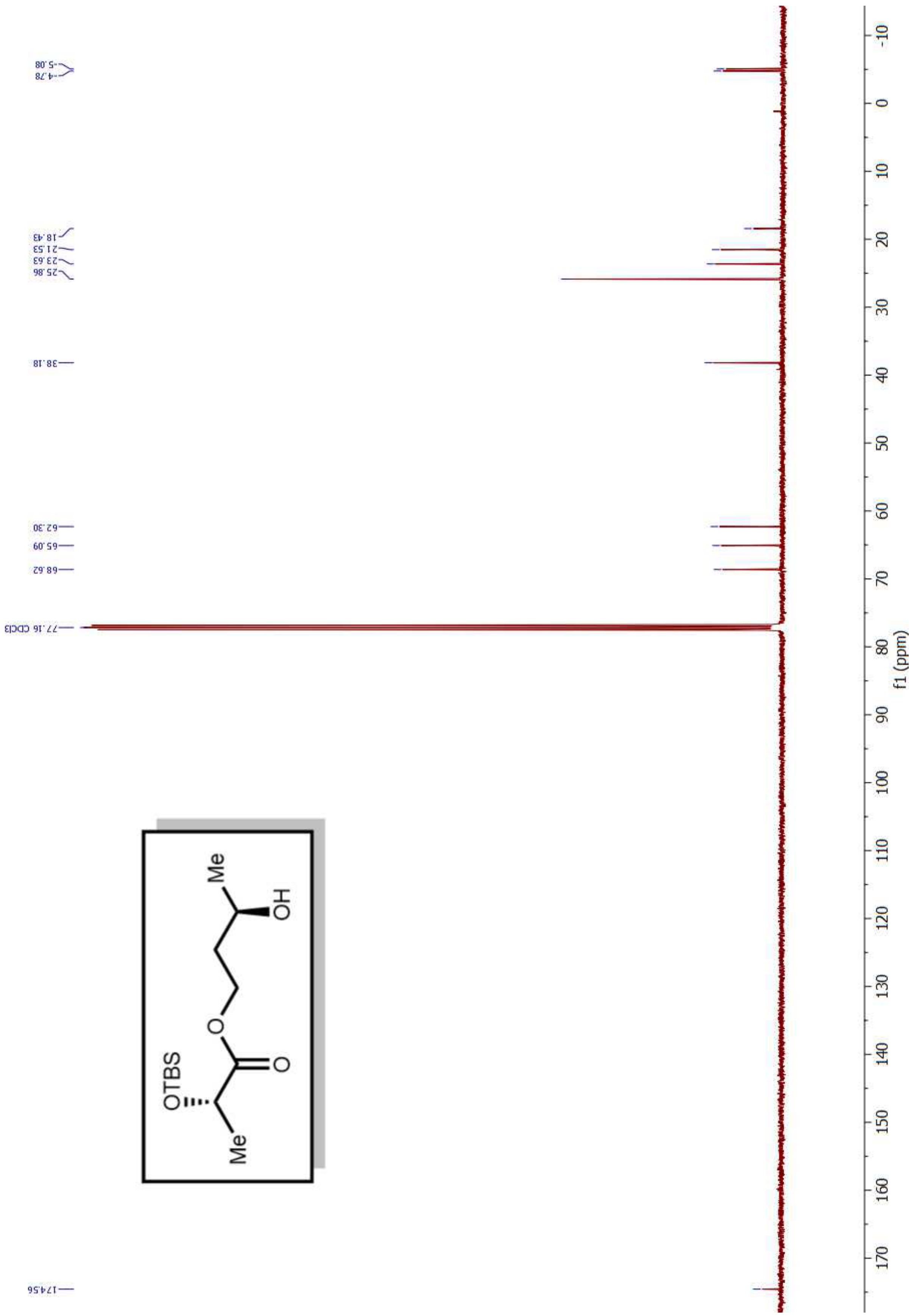
-5.08
-4.78
18.43
21.53
23.63
25.86
38.18
62.30
65.09
68.62
77.16 CDCl3
174.56
OTBS
Me
O
O
Me
OH
f1 (ppm)

Figure S6

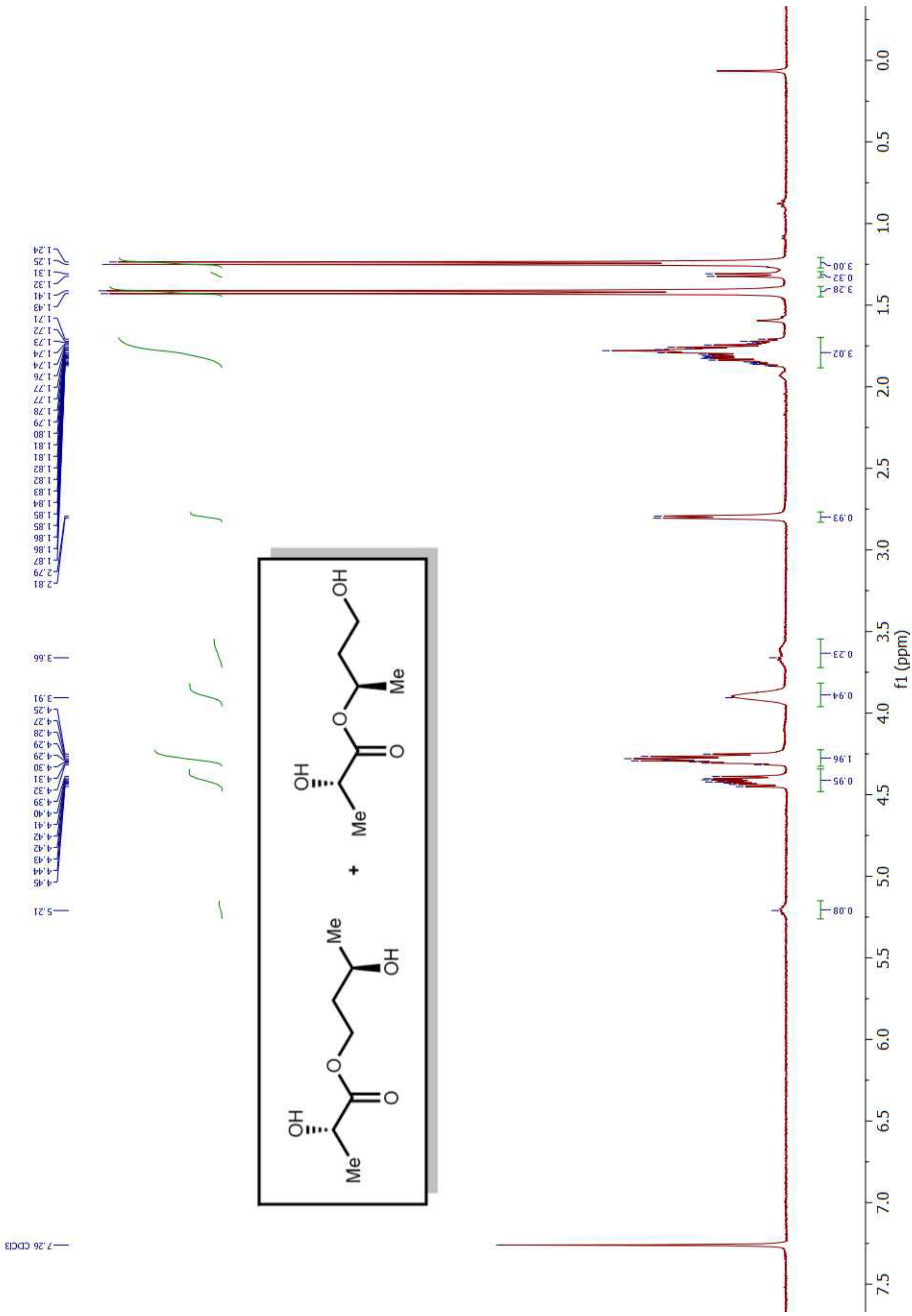

Figure S7

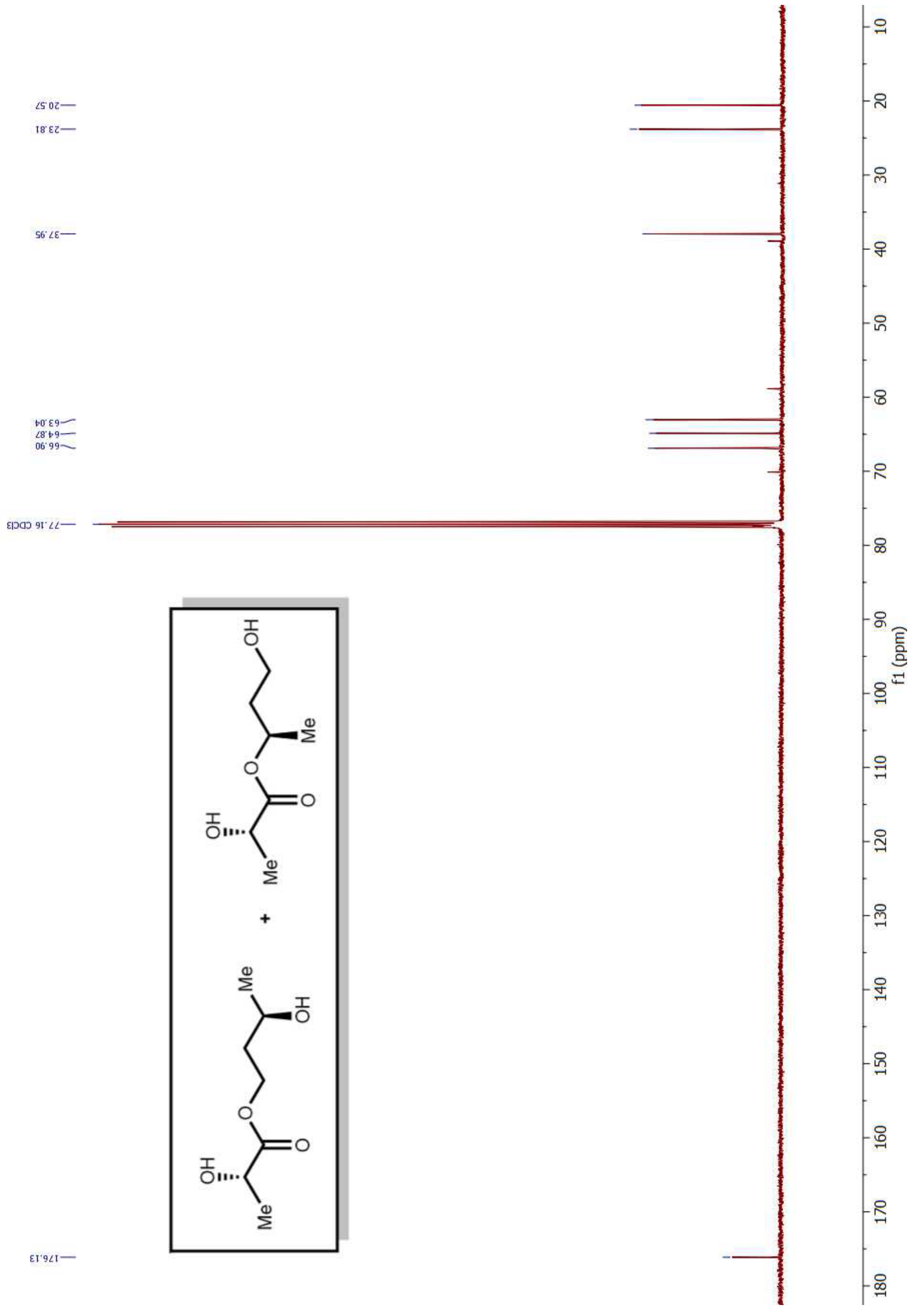

20.57
23.81
37.95
63.04
64.87
66.90
77.16 CDCl3
176.13
Me
OH
O
O
Me
OH
+
Me
OH
O
O
Me
OH
f1 (ppm)

Figure S8

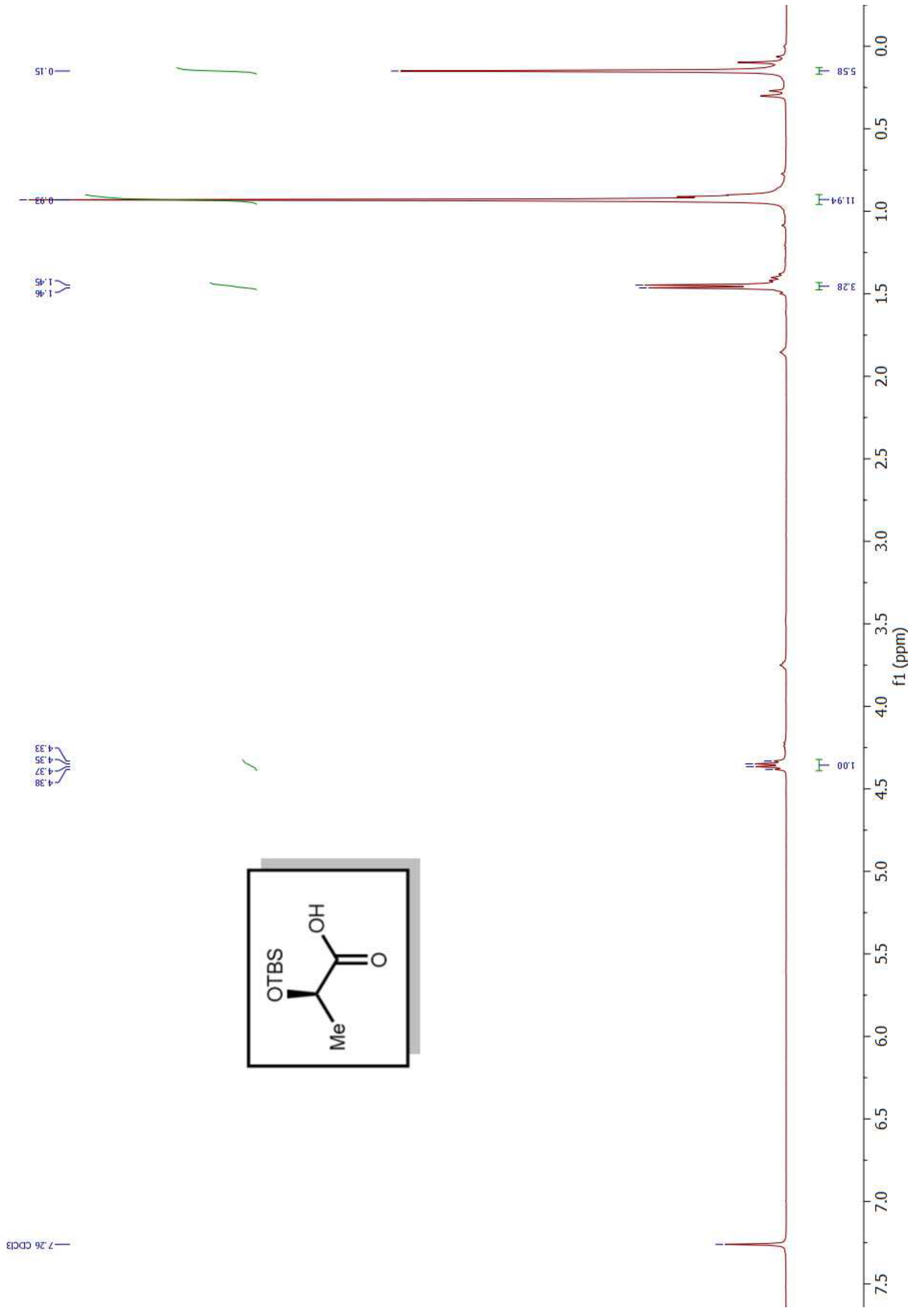

0.15
0.93
1.45
1.46
4.33
4.35
4.37
4.38
7.26 CDCl3
5.58
11.94
3.28
1.00
OTBS
OH
O
Me
f1 (ppm)

Figure S9

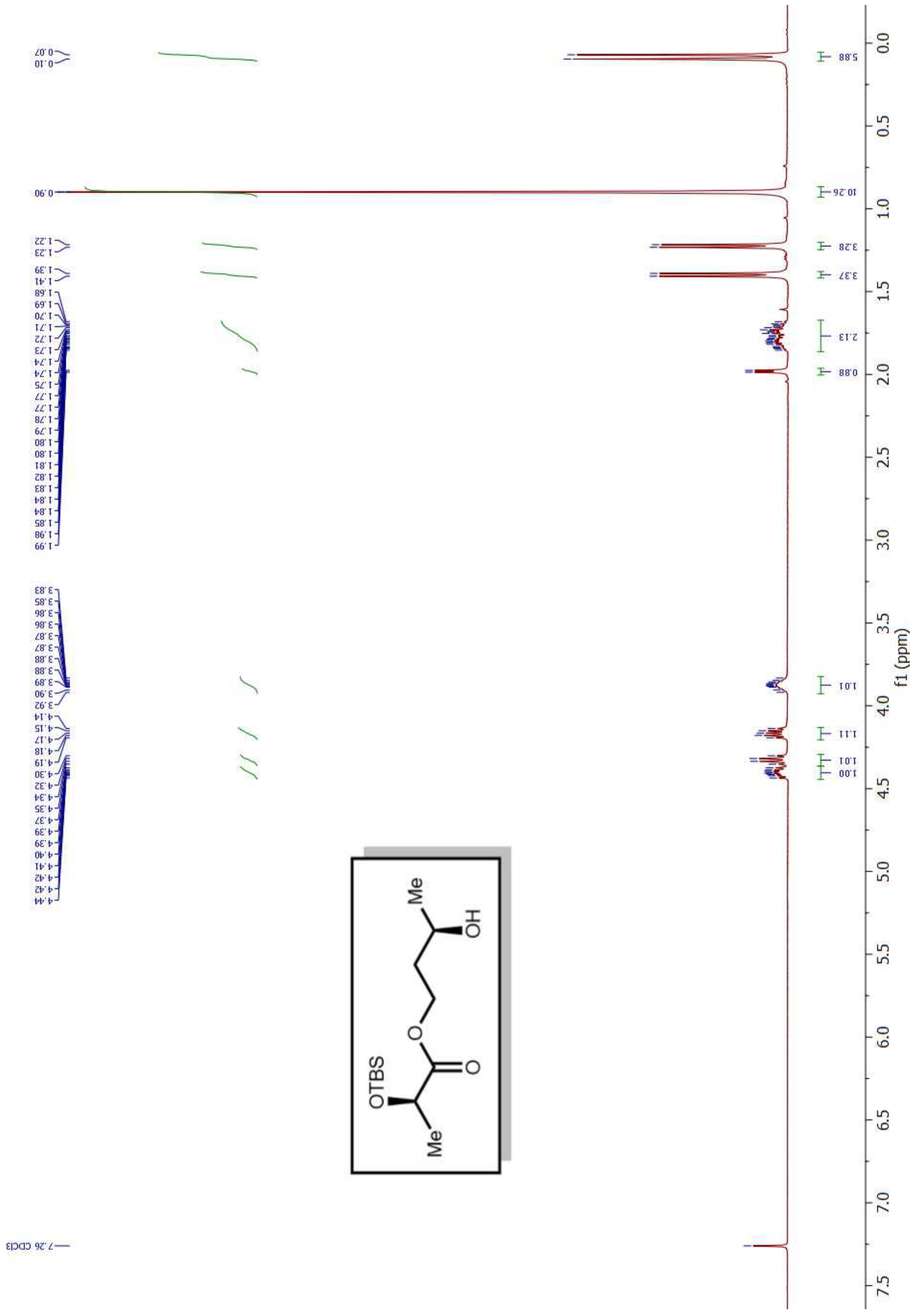

Figure S10

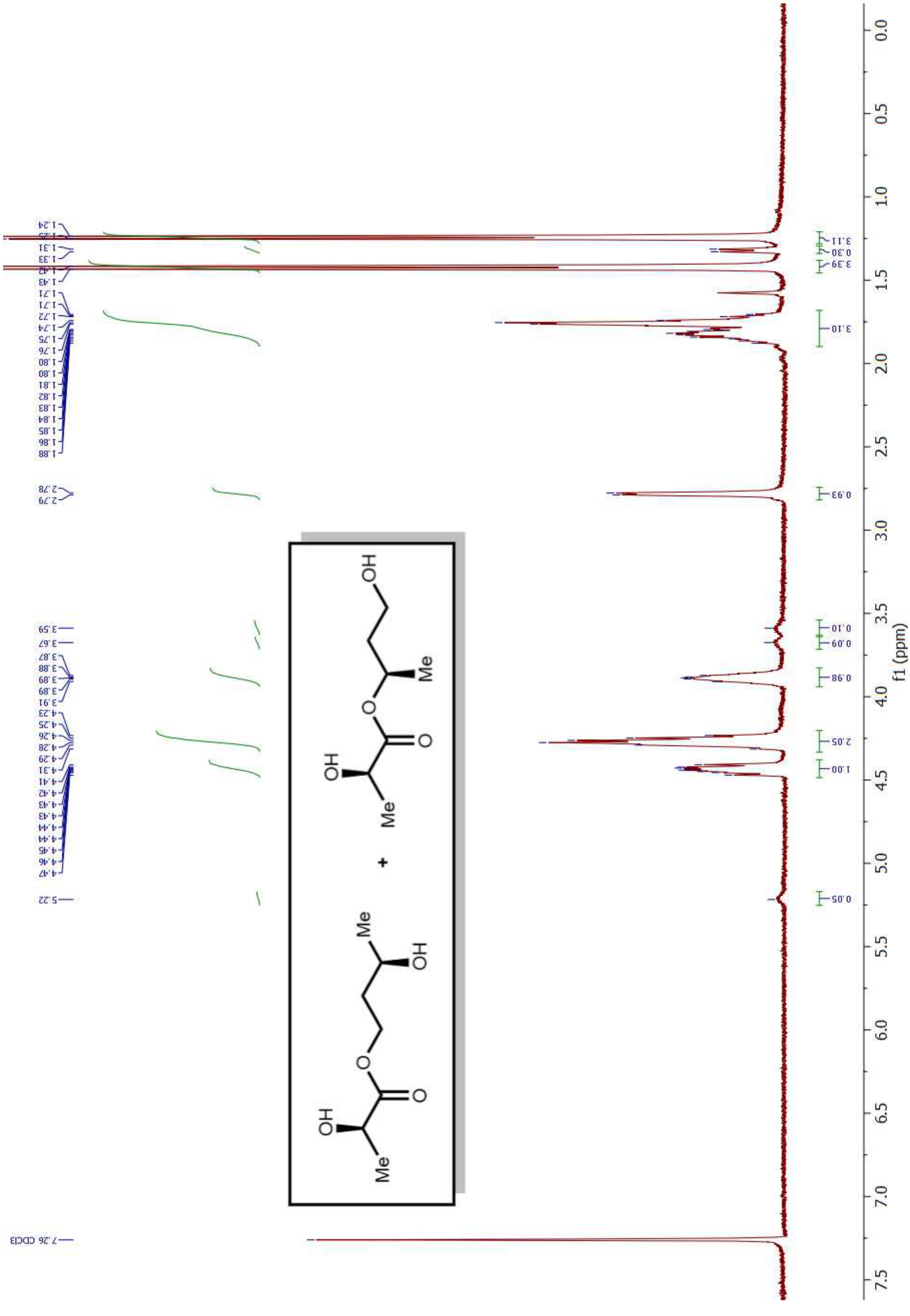

Figure S11

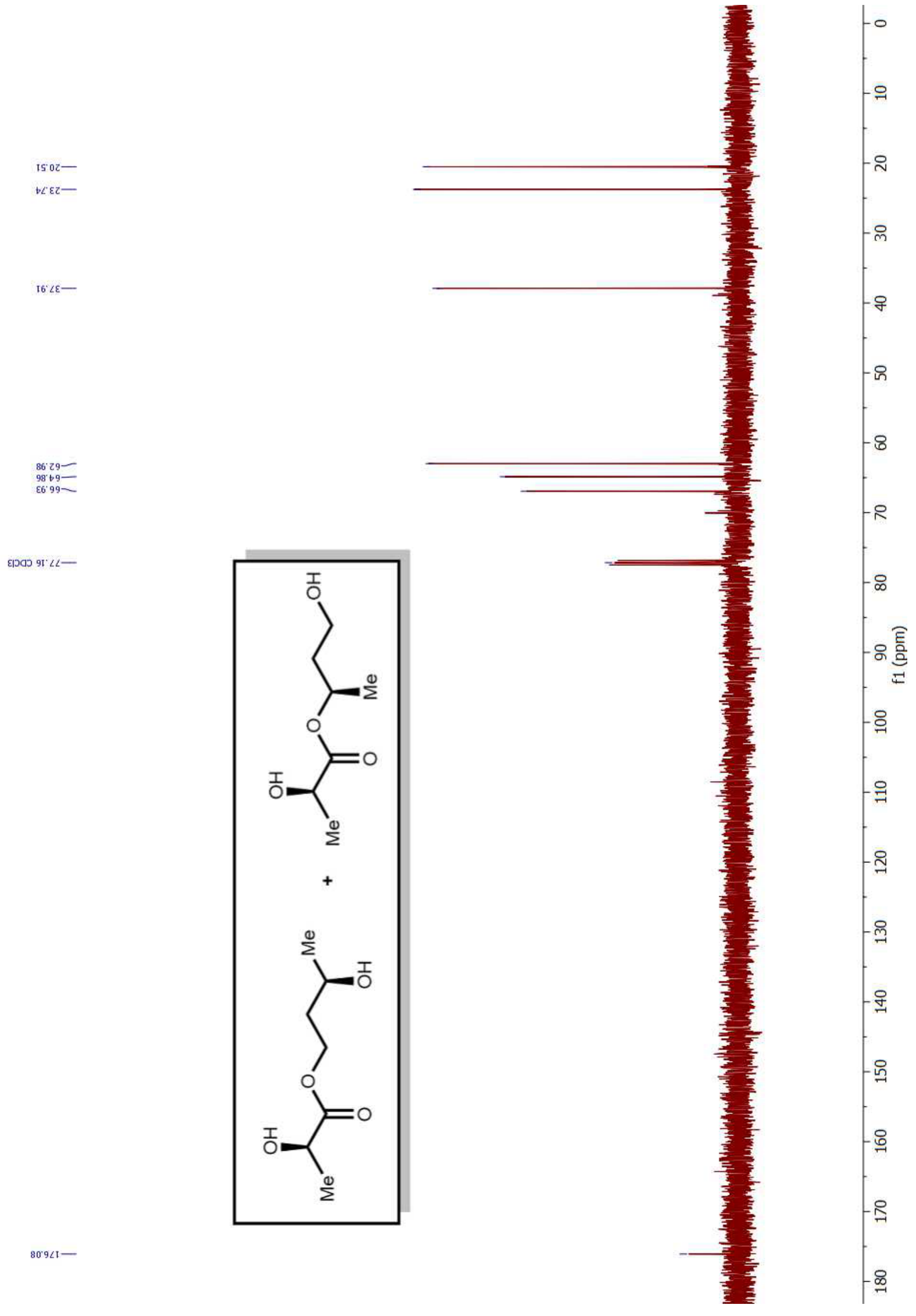

Figure S12
